# The crystal structure of the *Leishmania infantum* Silent Information Regulator 2 related protein 1: Implications to protein function and drug design

**DOI:** 10.1371/journal.pone.0193602

**Published:** 2018-03-15

**Authors:** Céline Ronin, David Mendes Costa, Joana Tavares, Joana Faria, Fabrice Ciesielski, Paola Ciapetti, Terry K. Smith, Jane MacDougall, Anabela Cordeiro-da-Silva, Iain K. Pemberton

**Affiliations:** 1 NovAliX - Bioparc, Bd Sébastien Brant, Illkirch, France; 2 i3S-Instituto de Investigação e Inovação em Saúde, Universidade do Porto, Porto, Portugal; 3 IBMC-, Instituto de Biologia Molecular e Celular da Universidade do Porto, Porto, Portuga; 4 BSRC, School of Biology, University of St Andrews, St Andrews, Scotland; 5 Photeomix, IP Research Consulting, Noisy Le Grand, France; 6 Departamento de Ciências Biológicas, Faculdade de Farmácia, Universidade do Porto, Porto, Portugal; George Washington University, UNITED STATES

## Abstract

The *de novo* crystal structure of the *Leishmania infantum* Silent Information Regulator 2 related protein 1 (LiSir2rp1) has been solved at 1.99Å in complex with an acetyl-lysine peptide substrate. The structure is broadly commensurate with Hst2/SIRT2 proteins of yeast and human origin, reproducing many of the structural features common to these sirtuin deacetylases, including the characteristic small zinc-binding domain, and the larger Rossmann-fold domain involved in NAD^+^-binding interactions. The two domains are linked via a cofactor binding loop ordered in open conformation. The peptide substrate binds to the LiSir2rp1 protein via a cleft formed between the small and large domains, with the acetyl-lysine side chain inserting further into the resultant hydrophobic tunnel. Crystals were obtained only with recombinant LiSir2rp1 possessing an extensive internal deletion of a proteolytically-sensitive region unique to the sirtuins of kinetoplastid origin. Deletion of 51 internal amino acids (P253-E303) from LiSir2rp1 did not appear to alter peptide substrate interactions in deacetylation assays, but was indispensable to obtain crystals. Removal of this potentially flexible region, that otherwise extends from the classical structural elements of the Rossmann-fold, specifically the β8-β9 connector, appears to result in lower accumulation of the protein when expressed from episomal vectors in *L*. *infantum* SIR2rp1 single knockout promastigotes. The biological function of the large serine-rich insertion in kinetoplastid/trypanosomatid sirtuins, highlighted as a disordered region with strong potential for post-translational modification, remains unknown but may confer additional cellular functions that are distinct from their human counterparts. These unique molecular features, along with the resolution of the first kinetoplastid sirtuin deacetylase structure, present novel opportunities for drug design against a protein target previously established as essential to parasite survival and proliferation.

## Introduction

Leishmaniasis is a neglected tropical disease caused by *Leishmania* parasites that affects millions of people worldwide, especially in tropical and subtropical areas, with high mortality and morbidity [1]. Visceral leishmaniasis, the most severe form of the disease, is invariably fatal if untreated. Disease control relies mostly on chemotherapy that has been associated with substantial safety issues and drug resistance amongst other shortcomings that have hindered disease eradication in endemic areas. The search for new, safe, and effective drugs remains an urgent, but as yet unmet need. The search for novel selective drug targets is therefore all the more imperative.

Protein acetylation has recently emerged as a major reversible post-translational modification where the (de)acetylation of the ε-amino group of lysine residues regulates the biological activities of a wide range of proteins and their associated cellular processes [2–4]. The modification was initially described in the N-terminal domain of histones, giving rise to the name of this superfamily of histone deacetylases (HDAC) of which four different classes exist based on sequence homology and domain organisation [3]. Histone acetylation in association with methylation, phosphorylation and other modifications regulates chromatin structure and the association of transcriptional factors with DNA [4]. Protein acetylation is now recognised to control many other cellular processes [2].

The proteins belonging to the Silent Information Regulator 2 (SIR2) family, also known as Sirtuins, are classified as class III HDAC proteins due to their dependency on NAD^+^ to deacetylate lysine residues of histones and non-histone substrates [5–7]. These proteins have been implicated in the regulation of a number of biological processes, such as gene silencing, DNA repair, longevity, metabolism, apoptosis, and development.

Sirtuins exhibit a remarkable level of structural and functional preservation throughout the six kingdoms of life. Structural alignment of sirtuins from diverse origins shows a high degree of structural conservation of the central catalytic core, comprising approximately 275 residues that confer the major acetyl lysine substrate and NAD^+^ cofactor binding functions [8]. Additional N- and C-terminal extensions of variable primary sequence and length contribute accessory functions that are specific to each individual class, though these remain poorly understood [9]. Sirtuins are generally composed of two domains, nominally (i) a Rossmann-fold domain, a structural signature common to all NAD(P)^+^-binding enzymes, and (ii) a smaller zinc-binding domain. Several discrete loops connect the two domains, the largest of which, the cofactor-binding loop, is subject to conformational modification upon NAD^+^ binding. The substrate binding cleft is formed at the interface between the two domains where a hydrophobic pocket, shaped by several residues adjacent to the cleft, accommodates the target acetyl group. Substrate binding involves several non-selective hydrogen bonding interactions with sirtuin main chain atoms, where a general lack of primary sequence specific interactions has been observed to date in co-crystal structures with peptide substrates. Conformational rather than sequence specificity appears to be a more important substrate determinant, which has been proposed to necessitate a region of intrinsic disorder to provide accessibility[10] and the capacity to adopt a required 3D conformation in the bound state, specifically a 3-stranded antiparallel β-sheet known as the β-staple, as observed for bacterial TmSIR2/peptide structures [11, 12] and hSIRT1 interactions with its pseudo-substrate [13].

Seven different classes of sirtuins, hSIRT1-7, exist in humans, whilst only three sirtuin homologues have been identified in the Trypanosomatids [14, 15], *Trypanosoma brucei* and *Leishmania* spp. have three sirtuins namely; SIR2 related protein 1 (SIR2rp1), SIR2rp2 and SIR2rp3. Some members of the SIR2 family like the yeast SIR2, the human SIRT2, the mouse SIRT6, the *T*. *brucei* SIR2rp1 and the *L*. *infantum* SIR2rp1 (LiSir2rp1) also exhibit ADP-ribosyltransferase activity [14, 16–19].

Phylogenetic analysis of 778 annotated sirtuins from 529 organisms subdivides these into five main clades, where LiSIR2rp1 belongs to class I along with hSIRT1-3 [20]. LiSIR2rp1 shows a high degree of sequence similarity with SIRT2 (46%), SIRT3(47%) and SIRT1 (42%) mainly due to conservation of the central catalytic core. The flanking N- and C-terminal regions are more variable in size and sequence and remain poorly characterised both structurally and functionally. The most extensive N and C-terminal additions seen in SIRT1 are mostly absent from LiSIR2rp1, which locates to the cytosol similar to hSIRT2, rather than the nucleus, as for hSIRT1 [20, 21].

In *Leishmania*, cytosolic SIR2rp1 presents NAD^+^-dependent deacetylase and ADP-ribosyltransferase activities unrelated to epigenetic silencing [21]. The importance of SIR2rp1 in *Leishmania* was elucidated with gene knockout studies. While single knockouts were readily obtainable, double deletion of the Sir2rp1 alleles in *L*. *infantum* was only possible after the rescue by an episomal copy of the gene, suggesting an essential role for parasite survival [22]. Moreover, when LiSIR2rp1 single-knockouts were used to infect macrophages, it was noted that although they had the same invasive capacity as wild-type parasites, they had a hindered replication rate leading to reduced infectivity over time. Furthermore, the mutated parasites failed to establish infection in an *in vivo* mouse model of leishmaniasis [22].

Interest has grown in sirtuins as potential targets for anti-parasitic drug development [15, 20]. *Leishmania* SIR2rp1 has emerged from the aforementioned studies as a promising drug target deserving exploitation, but prior to this work, the lack of structural information on this protein has hampered structure-based drug design. Despite the wealth of structural information available for sirtuins from varied prokaryotic and eukaryotic origins, a structure for the corresponding NAD^+^-dependent deacetylase from any of the protozoan kinetoplastid parasites has long been lacking. Here we describe the first high-resolution crystal structure of *Leishmania infantum* SIR2rp1.

## Materials and methods

### Reagents

Fluorescent acetylated peptides bearing C-terminal amidation were synthesized and HPLC purified to >95% purity for deacetylation assays (Genscript). TPCK Treated trypsin from bovine pancreas (≥10,000 BAEE units/mg protein) was obtained from Sigma Aldrich.

### Parasites

*L*. *infantum* (MHOM/MA/67/ITMAP-263) promastigotes were cultured in complete SDM or RPMI 1640 media as described previously [23]. The LiSIR2RP1 single knockout (neomycin resistant) parasites generated previously [22] were maintained in the appropriate selection medium [22].

#### Recombinant human SIRT2 protein production

Histidine-tagged human SIRT2 isoform 2 was produced by fusing the open reading frame sequence of the hSIRT2 protein (Uniprot Q81XJ6-2) to a N-terminal 6xHis tag in an in-houseT7 expression vector (pT7 HTST). The coding sequence was produced by gene synthesis after reverse translation of the hSIRT2 ORF to DNA *in silico* whilst accounting for the optimum GC content and codon usage bias in *E*. *coli* (Life Technologies SAS). The gene fragment was cloned into the *Xho* I and *BamH* I sites of pT7 HTST and the resultant sequence verified by DNA sequencing (Eurofins, Germany). Expression in *E*. *coli* BL21 (DE3) cells was performed by auto-induction overnight in LB supplemented with 100 μg/ml ampicillin and 0.1% lactose. Cells grown to an OD_600_ > 3 were harvested by centrifugation (3000 rpm for 20 min at 4°C) and resuspended in buffer A (50 mM Tris.HCl pH 8, 500 mM NaCl, 10 mM imidazole) supplemented with protease inhibitors (1x Complete^™^ EDTA-free protease inhibitors, Santa Cruz Biotechnology) prior to disruption by sonication (6 x 10 s using an MSE sonicator). Soluble proteins, obtained after centrifugation, were diluted 3-fold in buffer A, filtered through a 0.22 μm polyether sulfone membrane (Phenomenex) and loaded onto a 1 ml Ni^2+^-charged IMAC column (HisTrap, GE Heathcare) at 1 ml/min at 6°C. After washing with 30 ml of buffer A, hSIRT2 was eluted in buffer A + 100 mM imidazole. The eluate was desalted directly into storage buffer (50 mM Tris HCl pH 7.5, 50 mM NaCl, and 40% glycerol) using PD10 Sephadex columns according to manufacturer’s instructions (GE Healthcare).

### Recombinant LiSIR2rp1 protein production for proteolysis

Recombinant LiSIR2rp1 and associated deletion mutant proteins were expressed in *E*. *coli* and purified as 6xHis tagged proteins using standard techniques, essentially as described previously with minor modifications [21]. For proteolysis experiments, 6xHis LiSIR2rp1, and a LiSIR2rp1 ΔN mutant comprising amino acids 7–372 fused to the 6xHis tag, were expressed in *E*. *coli* BL21 (DE3) under the control of a T7 promoter prior to purification by Ni^2+^ -affinity chromatography, essentially as described above for hSIRT2.

### *In vitro* proteolysis

Native proteolysis was performed by incubating the recombinant LiSIR2rp1 protein (2 mg) with 0.02 mg TPCK treated trypsin (ratio 1/100) in a final volume of 4 ml buffer (50 mM Tris.HCl pH 8, 50 mM NaCl). Hydrolysis was performed for a specified time at 37°C before terminating proteolysis reactions with stop buffer (final concentration: 1 mM aminoethyl-benzene-sulfonyl fluoride, 0.1% w/v BSA) and analyzing samples on 20% SDS PAGE before or after re-purification on HisTrap columns. To analyze subunit interactions, HisTrap column washes were performed with the addition of 6 M urea. LC MS analysis of LiSIR2 native proteolysis reactions was performed without purification as described in S1 Text.

### Deacetylation assays

NAD^+^-dependent deacetylase activity was measured using an acetylated p53 peptide substrate (FITC-ahx-TSPQPKK-AC) and deacetylation monitored over time via a charge-based electrophoretic microfluidic mobility shift assay on a LabchipEZReader II (Perkin Elmer). For specific activity measurements, deacetylation was measured over several different protein concentrations at 2 min intervals in a reaction containing 50 mM Tris.HCl (pH 8), 5 mM MgCl_2_, 100 μg/ml BSA, 0.5 mM NAD^+^ and 20 μM peptide substrate in a final volume of 50 μl at 22°C. Specific activities (nmol/s/mg protein) were obtained by linear regression of initial reaction velocities (nmol/s) expressed against total protein (mg). For kinetic measurements, Km values were obtained at a fixed protein concentration (25 nM) by varying NAD^+^ (10–500μM) or peptide substrate (range: 1–100μM for Li*S*IR2rp1, 10–1000μM for hSIRT2). For peptide Km, the fluorescent peptide was mixed with varied concentrations of the equivalent non-fluorescent peptide to obtain the required concentration range whilst maintaining fixed levels of fluorescence between assays.

### Design of LiSIR2rp1 deletion constructs for the structural study

For deletion construct design, a detailed analysis of LiSIR2rp1 sequence (Uniprot #Q8I6E4) was carried out. Disordered regions and globular domain predictions were made with GlobPlot [24] and IUPred [25]. Secondary structure computed simulations were performed using Network Protein Sequence Analysis NPS@ [26] and PSIPRED web servers [27]. Multiple sequence alignments with yeast ScHst2 (Uniprot #P02309), human hSIRT1 (Uniprot #Q96EB6), hSIRT2 (Uniprot #Q8IXJ6) and hSIRT3 (Uniprot #Q9NTG7) homologues were calculated and analyzed with ClustalX [28]. LiSIR2rp1 homology 3D-models were generated using SWISS-MODEL [29] from structural data of closest homologues: yeast ScHst2 (pdb: 1q14, 1q17 and 1q1a) and human hSIRT2 (pdb: 3zgo and 1j8f).

### Protein production and purification for crystallization

LiSIR2rp1 deletion expression constructs were amplified by double-PCR (primers listed in S2 Table) and cloned into *Nde*I/*BamH*I sites of in-house modified pET-28b (thrombin cleavage site replaced by TEV cleavage site) and verified by DNA sequencing. Recombinant deletion mutants were produced in *E*. *coli* BL21 (DE3) as an N-terminal hexa-histidine tag with integrated Tobacco Etch Virus (TEV) protease site. Cells were grown in LB medium supplemented with kanamycin (50 μg/ml) at 37°C to an OD_600_ of 0.6. Protein expression was induced overnight at 18°C by adding isopropyl-thiogalactoside to a 0.7 mM final concentration. Bacterial cells were harvested by centrifugation and suspended in lysis buffer (50 mM TrisHCl pH7.5, 500 mM NaCl, 20 mM imidazole supplemented with complete^™^ EDTA-free Protease Inhibitor Cocktail (Roche Diagnostics). After sonication, the cell homogenate was centrifuged and the soluble tagged protein was purified by affinity chromatography on His60 Ni Superflow Resin (Clontech). Lysis buffer was used to wash the resin and the protein was eluted with buffer A (50 mM TrisHCl pH 7.5, 500 mM NaCl, 500 mM imidazole). Recombinant tobacco etch virus (TEV) was used to remove the tag (overnight, 4°C). Additional SEC was carried out on a HiLoad 26/60 Superdex 200 equilibrated with buffer B (50 mM TrisHCl pH7.5, 50 mM NaCl). Fractions containing the purified protein were pooled and the protein was concentrated to 15 mg/ml approximately.

### Crystallization

In the case of co-crystallization, purified LiSIR2rp1 deletion mutants were incubated with three molar equivalents of acetylated p53 peptide (372-KKGQSTSRHK-K[Ac]-LMFKTEG-389). Crystallization experiments were carried out using the sitting drop vapor diffusion method in 96-well plates using an Innovadyne nanodrop robot. Crystals were grown in a drop composed of 300 nl protein/p53 mixture and 300nl of reservoir solution (100 mM Hepes pH7.5 and 25% PEG2000 MME) at 22°C.

### Data collection and structure determination

Crystals were cryo-cooled in liquid nitrogen in the crystallization condition supplemented with 22% glycerol. Diffraction data were collected on Proxima 1 beamline (SOLEIL, Saclay, France) on a Pilatus 6M detector. Data were processed with xds [30] and CCP4 software package [31]. Structure was solved using the automated molecular replacement pipeline, Mr Bump [32]. The path that led to a successful solution employed MOLREP [33] for molecular replacement and a search model prepared by CHAINSAW [34] from human SIRT3 homologue (pdb: 3glt). Model building and improvement were conducted by iterative cycles of manual building with Coot [35] and refinement with Refmac [36] (Table 1). The quality of the model was monitored with Rampage (integrated in CCP4 Suite) and final structure validated using the PDB validation server. The final model includes two LiSIR2rp1(ΔP253-E303) molecules in the asymmetric unit. For each LiSIR2rp1(ΔP253-E303) molecule, inspection of the Fo-Fc map revealed unmodeled contiguous density in the cleft between the two domains of the protein that could be attributed to part of the acetylated p53 peptide ((S)-RHK-K[Ac]-LMFK). The PYMOL Molecular Graphics System [37] was used for Rmsd and domain shift calculations as well as the generation of molecular images except where the UCSF Chimera package was used [38].

**Table 1:** Data collection and refinement statistics.

Complex PDB Id*	LiSIR2rp1(ΔP253-E303) /p53- K[Ac] 5OL0
**Data collection**	
Resolution (Å)	46.6–2.0 (2.1–2.0)‡
Space group	P2_1_
Unit-cell parameters	
a, b, c (Å)	a = 66.0, b = 65.6, c = 66.6
α, β, γ (°)	α = γ = 90 β = 97.7
Completeness (%)	98.8 (95.4)
Redundancy	3.4 (3.1)
I/σ(I)	15.4 (2.2)
Rsym(I) (%)	4.7 (50.6)
**Refinement**	
R_work_ (%)	16.7 (27.2)
R_free_ (%)	22.6 (33.0)
rmsd in bond lengths (Å)	0.02
rmsd in bond angles (°)	1.83
Complex / A.U.	2
No. atoms /complex	(Protein/peptide/Zn/solvent)
chains A/C	2190 / 78 / 1 / 117
chains B/D	2033 / 84 / 1 / 123
Mean B factors (Å^2^)	Complex (Protein/peptide/Zn/solvent)
chains A/C	42.6 (42.1/41.8/53.7/44.4)
chains B/D	41.9 (40.7/47.2/58.6/45.1)
Ramachandran Plot (Rampage)	
Favoured (%)	97.4
Allowed (%)	2.4
Outlier (%)	0.2

^‡^ Values in parentheses refer to the last resolution shell

* Structure data can be found at https://www.rcsb.org/ under accession code 5OL0

### Protein disorder analysis

In addition to predictions made during deletion constructions, protein disorder was further analysed by DisEMBL, a web-based tool complementary to GlobPlot [24] that implements 3 different algorithms [39]. As the definitions of protein disorder vary fundamentally between these various tools, different prediction tools give different results that should be corroborated with experimental evidence. In this report, we favored the Remark465 definition, an artificial neural network trained on the coordinates missing from structures deposited in pdb, most likely due to intrinsic disorder. Whilst the Remark465 and hot-loops definitions gave relatively similar results and are generally consistent with the structural data obtained in this report, the loops/coil definition generated several false IDRs.

### Generation of LiSIR2rp1 promastigote expression constructs

The open reading frame of *Li*SIR2rp1 and the sequences encoding the previously cloned truncated forms were amplified by PCR using a Taq DNA polymerase with proofreading activity (Roche). The amplicons were cloned into pGEM-T Easy Vector (Promega), sequenced and then subcloned into the *Xba*I/*Nde*I site of pSP72αBLASTα vector.

### Complementation of LiSIR2rp1 sKO promastigotes with pSPαBLASTα*Li*SIR2RP1 constructs

Fifty million mid-log LiSIR2rp1 sKO promastigotes were transfected with a Nucleofector II Device (Amaxa) using approximately 5 μg of each pSPαBLASTα*Li*SIR2RP1 plasmid and the Human T-Cell Nucleofector kit (Lonza). Following electroporation, parasites were incubated at 26°C for 24 h in complete SDM medium. Blasticidin was added to the cultures at 30 μg/mL and parasites were subcultured under drug pressure for 5 weeks before being used in experiments.

### SDS-PAGE and Western blot analysis

Five μg proteins were run on a 12% SDS-PAGE gel, followed by Coomassie blue staining. For Western blot, 100 ng of recombinant protein or parasite extracts corresponding to 2x10^7^ stationary promastigotes were used and transfer onto a nitrocellulose membrane using a Trans-Blot Turbo Transfer System (Bio-Rad) ensued. The membrane was blocked with 5% (w/v) non-fat dried skimmed milk in PBS/0.1% Tween 20 (1 h at RT), washed, incubated with primary antibody, washed, incubated with secondary antibody and washed again. Washes were performed at RT using PBS/0.1% Tween 20 (once for 15 min and 3 times for 5 min). Incubation conditions were the following: mouse anti-*Lm*SIR2RP1 IIIG4 mAb [40], overnight at 4°C or 2 h at RT; horseradish peroxidase (HRP)-conjugated goat anti-mouse IgG (1:10000) (Southern Biotech), 1 h at RT; rabbit anti-*Lm*CS (1:2000), 1.5 h at RT; HRP-conjugated goat anti-rabbit IgG (1:5000) (Southern Biotech), 1 h at RT. The membranes were then developed using SuperSignal WestPico Chemiluminescent Substrate (Thermo Scientific). Parasite extracts were prepared by boiling the samples in 2X SDS-PAGE loading buffer for 5 min.

### Real-time quantitative PCR analysis

To determine the expression levels of LiSIR2rp1 and the blasticidin selectable marker in promastigotes, RNA was extracted using approximately 2x10^8^ parasites and TRIzol reagent (Invitrogen). Reverse transcription for cDNA production was performed using the NZY First-Strand cDNA Synthesis Kit (NZYtech). Appropriate dilutions of cDNA and primers 3 + 4 and 5 + 6 (S3 Table) were used in the reactions for LiSIR2rp1 and blasticidin resistance marker open reading frames, respectively. To assess the levels of pSPαBLASTα LiSIR2rp1 constructs, genomic DNA extraction was performed using at least 1.5x10^8^ promastigotes and DNAzol reagent (Invitrogen). Ten ng of genomic DNA and primers 7 + 8 (S3 Table) were used in each reaction. In either case, *rRNA45* was used as the reference gene with primers 9 + 10 [41]. Amplification efficiencies were determined and the Pfaffl method [42] was employed to calculate expression or exponential ratios.

## Results

### Direct evidence of a proteolytically-sensitive flexible region linking tightly associated LiSIR2rp1 N- and C-terminal regions

**Fig 1:**
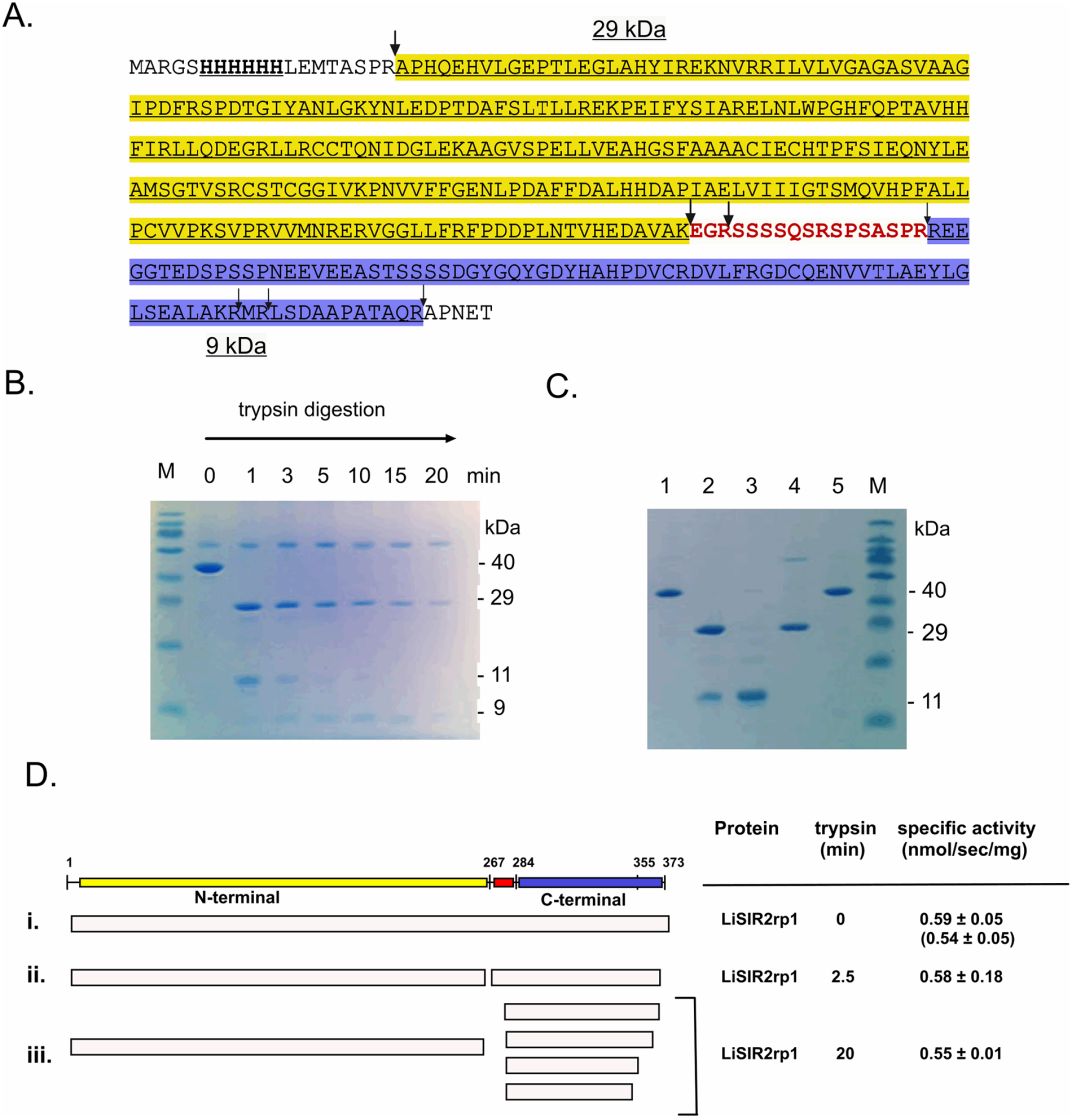
Probing the proteolytically-stable domain structure of LiSIR2rp1 and its deacetylase activity. **(A)** Primary amino acid sequence of 6 x His N-terminal tagged LiSIR2rp1 showing positions sensitive to tryptic digestion under non-denaturing conditions (arrows), defined from SDS PAGE and LC/ESI-TOF MS (see Methods and S1 Text). The larger arrows indicate principal digestion sites in time-resolved proteolysis. The proteolytically-sensitive region linking proteolytically-stable N-terminal (yellow) and C-terminal (blue) regions is shown in red. **(B)** Time-resolved tryptic digestion (0–20 min) of LiSIR2rp1 showing appearance of an N-terminal, ~29 kDa region (28580.83 and 28921.53 Da) and a ~ 9 kDa C-terminal region (9593.98, 9081.4, 7999.25 and 7710.43 Da) derived from an initial ~ 11 kDa digestion product (t = 1 to 3 min). (*The larger protein at 66 kDa is albumin in the quenching buffer*). **(C)** Re-purification of His tagged LiSIR2rp1 after native tryptic digestion; a 6 x His LiSIR2rp1 ΔN^5-373^ mutant protein lacking the N-terminal trypsin site was used for these experiments (see Methods). **Lanes 1** and **5**, undigested LiSIR2rp1 purified on Ni^2+^- affinity column. **Lane 2**, native digest of LiSIR2rp1 re-purified on Ni^2+^-affinity column (N- and C- termini bind). **Lane 3**, 6 M urea wash of native digest of LiSIR2rp1 bound to Ni^2+^- affinity column (C-terminal domain removed). **Lane 4**, Elution of native digest from Ni^2+^-affinity column with imidazole after 6 M urea wash (N-terminus alone). **(D)** Sirtuin deacetylase activity of LiSIR2rp1 after native tryptic digestion. Time resolved digests of 6 x His LiSIR2rp1 ΔN^5-373^ were re-purified from trypsin reactions by Ni^2+^- affinity chromatography before assessing specific activity on the p53 acetyl lysine substrate. Specific activity (nmol deacetylated product/sec/mg protein) was compared for (i) undigested protein (t = 0) measured both for 6xhis LiSIR2rp1 ΔN^5-373^ and full length protein (shown in parentheses), (ii) a 2.5 min digest and (iii) a 20 min digest. The fragmentation patterns were deduced from the results of SDS PAGE and LC/MS analyses.

Recombinant LiSir2rp1 is a soluble, biochemically active protein available in large quantities [21]. Despite extensive crystallization screening on the full length LiSIR2rp1 protein, either alone or with varied p53 peptide substrate and/or NAD^+^ combinations, all attempts to obtain crystals proved unsuccessful. Highly flexible and dynamically disordered regions are commonplace amongst sirtuin family members from a wide number of organisms [9], in addition to being prevalent within intractable proteins that fail to produce satisfactory crystals for structure determinations [43]. Consequently, native proteolytic digestion of the recombinant protein was used to probe for the presence of stable subdomains and/or intrinsically disordered regions that may interfere with crystal formation. Local unfolding of at least 13 residues is required for a protein region to fit productively into the active site of trypsin and serve as a proteolytic substrate [44, 45]. The rate and extent of proteolytic digestion is thereby predicted to reflect not only surface exposure but also the relative flexibility of the region located in the vicinity of the trypsin site, following previously established experimental approaches [43–45]. Time-controlled digestion of LiSIR2rp1 with trypsin under native conditions, and subsequent analysis by SDS PAGE and LC/ESI-TOF MS, revealed the presence of two principal proteolytically-stable polypeptide fragments (Fig 1 and S1 Text).

The major trypsin cleavage sites inferred by these data are mapped to the primary sequence in Fig 1A. These were confirmed by SDS PAGE (Fig 1B) and mass spectrometry (see S1 Text) to yield proteolytically stable regions consisting of a 29 kDa N-terminal fragment and an initial 11 kDa C-terminal fragment that is subsequently proteolysed to smaller stable fragments (approx. 7–9 kDa). The two domains remain tightly associated under native conditions upon tryptic digestion, but are readily dissociated upon denaturation with 6M urea (Fig 1C). These results suggest LiSir2rp1 is composed of two tightly associated proteolytically stable polypeptides (nominally 29 kDa N-terminal and 9 kDa C-terminal) linked minimally by a 17 residue surface-exposed, serine-rich peptide region 268–284, EGRSSSSQSRSPSASPR.

The rapid digestion at the internal tryptic site (nominally K267 or R270—S1 Text) was monitored more closely in order to assess the relative accessibility of this region to trypsin (S1 Fig). The temporal appearance of the stable ~29 kDa fragment (A6-K267/R270 –S1 Text) is initially preceded by a short-lived larger ~31 kDa fragment that retains the hexa-histidine tag and other residues preceding R6 (S1 Fig). The initial rapid digestion at K267/R270 is highly suggestive of conformational flexibility in the vicinity since sufficient local unwinding or disorder must occur to allow rapid association with the active site of trypsin [44]. Indeed, whilst the primary tryptic cleavage event at K267/R270 approaches complete digestion in less than 6 s (t_1/2_ ~ 1.5 s), R6 is proteolysed more slowly displaying a half-life of 20 ± 5 s (n = 3), despite its presence within an N-terminal region predicted to be disordered (see S1 Fig). In comparison, the 29 kDa fragment is considerably more stable exhibiting a half-life of many minutes (Fig 1B and S1 Fig).

The protein obtained from tryptic digests performed under native conditions retains full activity in deacetylation assays (Fig 1D), indicating that the intervening amino acids are dispensable for this function (in addition to residues trimmed from the N- and C- termini respectively). However, neither domain was soluble when expressed alone in *Escherichia coli* as recombinant protein, precluding their direct purification for crystallization studies.

### The proteolytically sensitive region aligns with a potentially disordered and poorly conserved insertion region in LiSir2rp1

**Fig 2:**
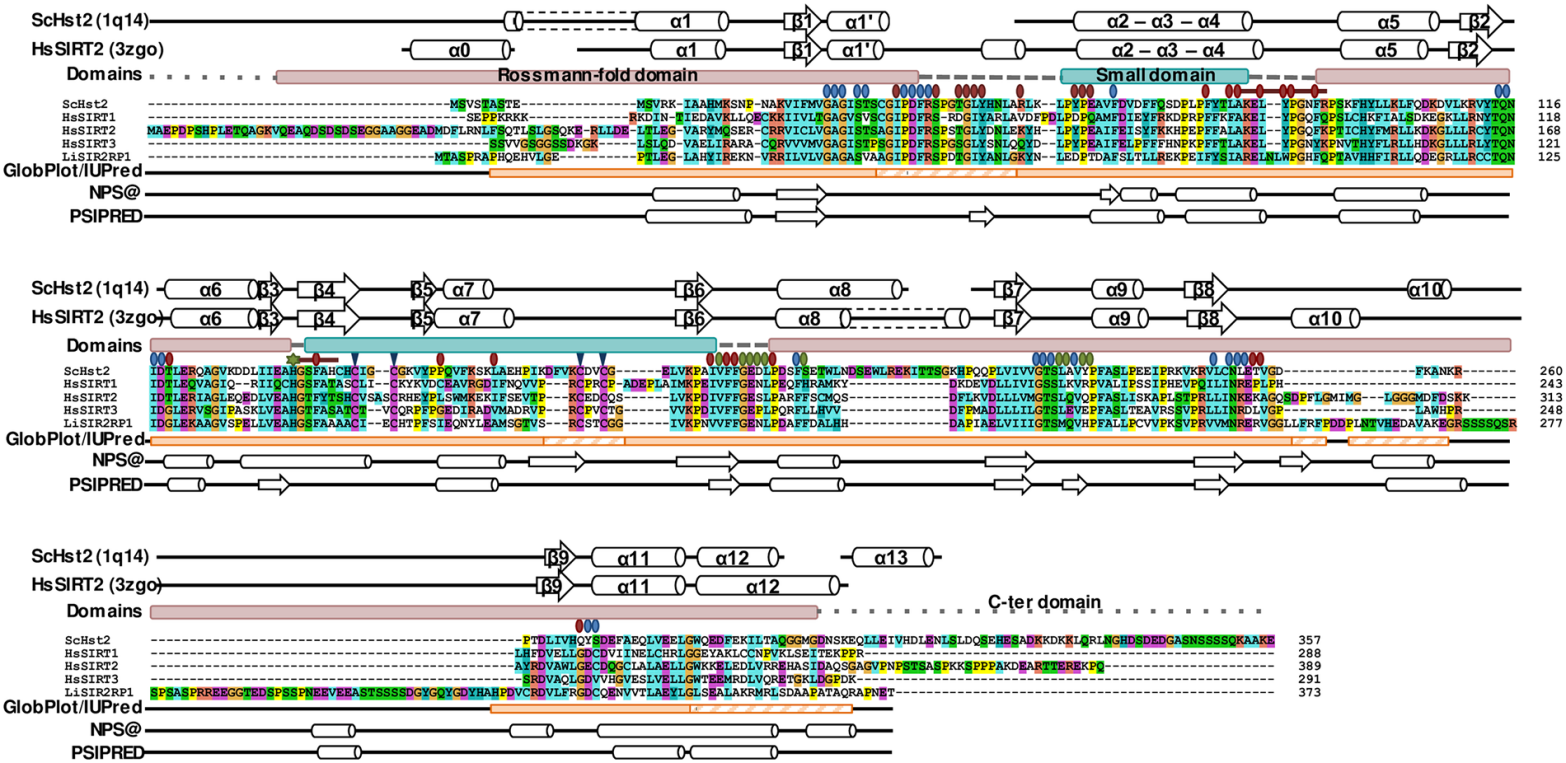
Multiple sequence alignments of LiSIR2RP1 with yeast Hst2 and human SIRT1-3 homologues. Multiple sequence alignment of LiSIR2rp1 (Uniprot #Q8I6E4) with ScHst2 (P53686), hSIRT1 (Q96EB6), hSIRT2 (Q8IXJ6) and hSIRT3 (Q9NTG7) (ScHst2 region 1–228 and 517–747 and hSIRT3 1–108 were omitted for clarity). Above the alignment, boxes define the Rossmann-fold (pink), and the small domain (blue). The main secondary structure elements described in ScHst2 (pdb: 1q14) and hSIRT2(pdb: 3zgo) are indicated. Important residues are pointed: residues important for binding of the NAD (blue circle), acetyl-lysine peptide (green circle) and Zinc ion (blue triangle) and identified as hot-spot by Parenti *et al*. (red circle) [56]. ECS site and selectivity binding pocket are delimited by a red line. Results of globular domain predictions by GlobPlot and IUPred are depicted as orange boxes (full colored in case of consensus between both algorithm and hachured when identified by only one). Results of secondary structure predictions by NPS@ and PSIPRED are represented beneath (β sheets are indicated with arrows and α helices with cylinders).

The paucity of arginine and lysine residues flanking the 17 amino acid linker prevented us from defining the full extent of the proteolytically sensitive and ostensively structurally-disordered region. Multiple sequence alignments show this region to be poorly conserved amongst SIR2 (SIRT2) proteins, where it coincides with a large insertion in LiSIR2rp1 spanning approximately 76 amino acids (Fig 2). The serine-rich insertion, F250-V325, situated between the β8 and β9-strands of previously solved structures [8], is absent in the human SIRT2 and yeast Hst2 proteins and thus appears to be an unusual structural addition to the classical Rossmann-fold (Fig 2). Interestingly, even the smaller β8- β9 connector region is considered to be a hSIRT2 specific insertion when aligned with other sirtuin classes (SIRT1, SIRT3), where structural studies show it to exist as an α-helix in apo conformation or an unwound loop upon substrate and cofactor binding [46, 47]. In the latter case, the loop appears to act as a pseudo substrate in crystal dimers with a leucine (Leu297) occupying the acetyl lysine binding site of the neighboring SIRT2 molecule [46, 47]. Structural algorithms generate little evidence for secondary structure elements within this region of LiSIR2rp1, predicted to be disordered/non-globular (see Methods). Furthermore, protein homology structure modeling (SWISS MODEL) [29], based upon the3D structures of the human SIRT2 (pdb: 3zgo) and yeast Hst2 (pdb: 1q14) homologues, predicts the intervening β8-β9 connector insertion to be present as a long unstructured ‘loop’ in several models (S2 Fig). Whilst this does not preclude the existence of this region as an independent domain, further structural folding through post-translational modifications and/or protein-protein interactions may be required to promote conformational stability in parasite cells.

This large insertion is absent from previously crystallized homologues [47–52]. Consequently, the region, along with its potential for disorder and/or interference with crystal formation, was removed in a number of specifically-designed deletion constructs.

### β8-β9 connector deletion mutants retain NAD dependent deacetylase function

Targeted deletion mutants were constructed to determine the impact of removing defined portions of the large β8-β9 intervening region on NAD-dependent protein deacetylase function. Initial screens confirmed that the LiSIR2rp1 mutants ΔL257-S282, ΔL257-S293, ΔL257-E303, and ΔP253-E303 each retained deacetylase activity towards the p53 acetyl -lysine substrate. In Table 2, the specific activities and associated enzyme parameters obtained for two such deletion mutant proteins are compared to full length LiSIR2rp1 and the human homologue, hSIRT2. The mutants were chosen as they represent the smallest and the largest deletions created for initial analyses (26 and 51 residues respectively). Deletion of 26 internal amino acids in the LiSIR2rp1 ΔL257-S282 mutant did not alter the Michaelis constant, Km, substantially for the essential cofactor, NAD^+^ when compared to the full length protein (88.9 ± 1.1 μM versus 99.6 ± 3.8 μM respectively); the values also remained similar to that obtained for human SIRT2 (Km^NAD+^ = 86.1 ± 12.7 μM). Similarly, LiSIR2rp1 ΔL257-S282 and ΔP253-E303 each retained Km values for the p53 acetyl-lysine substrate close to that of the full-length enzyme (Table 1). In this case, Km^p53K(Ac)^ values for both mutant and full length LiSir2rp1 proteins were ~4-fold lower that obtained with human SIRT2 (75.1 ± 13.7 μM).

**Table 2:** NAD^+^-dependent deacetylase activity of recombinant LiSIR2rp1 protein with directed internal deletions within the β8-β9 connector.

Deletion mutant	Deletion Length (aa)	Specific Activity^(2)^ (nmol/s/mg)	Km^NAD+^ (μM)	Km^p53K(Ac)^ (μM)	kcat (s^-1^)
LiSir2rp1^(1)^	0	0.59 ± 0.34 (n = 21)	99.6 ± 3.8	22.9 ± 4.0	0.21
LiSir2rp1^ΔL257-S282^	26	0.39 ± 0.15 (n = 3)^(3)^	88.9 ± 1.1	22.8 ± 5.0	0.10
LiSir2rp1^ΔP253-E303^	51	0.34 ± 0.06 (n = 2)	127.1 ± 28	15.6 ± 3.8	0.07
hSIRT2^(1)^	0	1.10 ± 0.24 (n = 5)	86.1± 12.7	75.1 ± 13.7	0.25

aa: amino acids, nd: not determined

^(1)^ Full length protein

^(2)^ Average ± SD

^(3)^ Not statistically significant when compared to LiSir2rp1 (unpaired student t-tests, P > 0.05).

In agreement with earlier proteolysis experiments (described above), these results indicate that large stretches of the β8-β9 connector (up to 51 amino acids) can be removed in the expressed protein without disrupting the NAD^+^-dependent protein deacetylase function. Furthermore, the specific deletions did not alter the final purification yield or purity. Whilst these experiments appear to reveal a moderate reduction in specific activities for the two deletion mutants, the average specific activities remain within the standard deviation limits measured for 21 different purifications of full length LiSIR2rp1 (0.59 ± 0.34 nmol/s/mg). Since no significant alteration in specific activity was observed previously for LiSIR2rp1 after portions of the loop were removed by trypsin (Fig 1D), these observations might suggest that the experimental variations in specific activities (and therefore kcat) may be a consequence of subtle differences in the individual preparations of the recombinant proteins rather than a *bona fide* minor alteration to catalytic efficiency.

### Purification, crystallization and structure determination of LiSIR2rp1 deletion mutants

Four different internal deletion mutants (LiSir2rp1 ΔP253-E303, ΔP253-H322, ΔS272-S310 and ΔS272-H322 –see S2 Text for details on deletion mutants design) were included in the structural study. All four recombinant mutants could be expressed and purified with similar final yield (≈10-15mg of purified protein recovered from 1L of bacterial culture) and show high stability in solution, suggesting extensive internal deletion did not impair proper global folding of the protein. Therefore, all the LiSIR2rp1 deletion mutants were tested for crystallization, alone or with acetylated p53 peptide substrate with or without NAD^+^ cofactor. Crystals suitable for X-ray diffraction could be obtained only with LiSIR2rp1(ΔP253-E303) mutant, uniquely in the presence of the p53 peptide substrate.

The LiSIR2rp1 (ΔP253-E303) structure was solved by molecular replacement. As a first attempt, yeast ScHst2 [49], human hSIRT2 [47] and LiSIR2rp1(ΔP253-E303) 3D-homology structures were used as search models. All led to unsatisfactory solution (R/Rfree higher than 50%). In a second attempt, automated molecular replacement pipelines Balbes [53] and MrBump [32] were used. Interestingly, in both cases, best solutions were based on human SIRT3: apo protein structure (pdb: 3gls) and protein/AceCS2K/ADPR complex (pdb: 3glt) [54] for Balbes and MrBump, respectively. Preliminary refinement, revealed the latter to be the best initial model for LiSIR2rp1 structure determination. After several iterative cycles of manual building and refinement, the structure of the LiSIR2rp1(ΔP253-E303)/p53 complex was solved at 1.99Å resolution (Pdb: 5Ol0). Crystals belonged to the P2_1_ space group and contained two LiSIR2rp1(ΔP253-E303) /p53 complexes per asymmetric unit.

In addition to a small number of residues missing at both N- and C- terminal ends of the protein (N-ter M1-R6 and C-ter A371-T373 are missing in both monomers), region R52-P70 could not be modeled in monomer B. Moreover, additionally to region P253-E303 removed from the recombinant protein, electron density was missing for residues A304 to P323 in both monomers, suggesting potential flexibility of this region. This makes us confident that deletion of P253-E303 region did not impair LiSIR2rp1 proper tertiary folding by introducing artificial constraints.

### Structure of the LiSir2rp1(ΔP253-E303)/p53 complex

**Fig 3:**
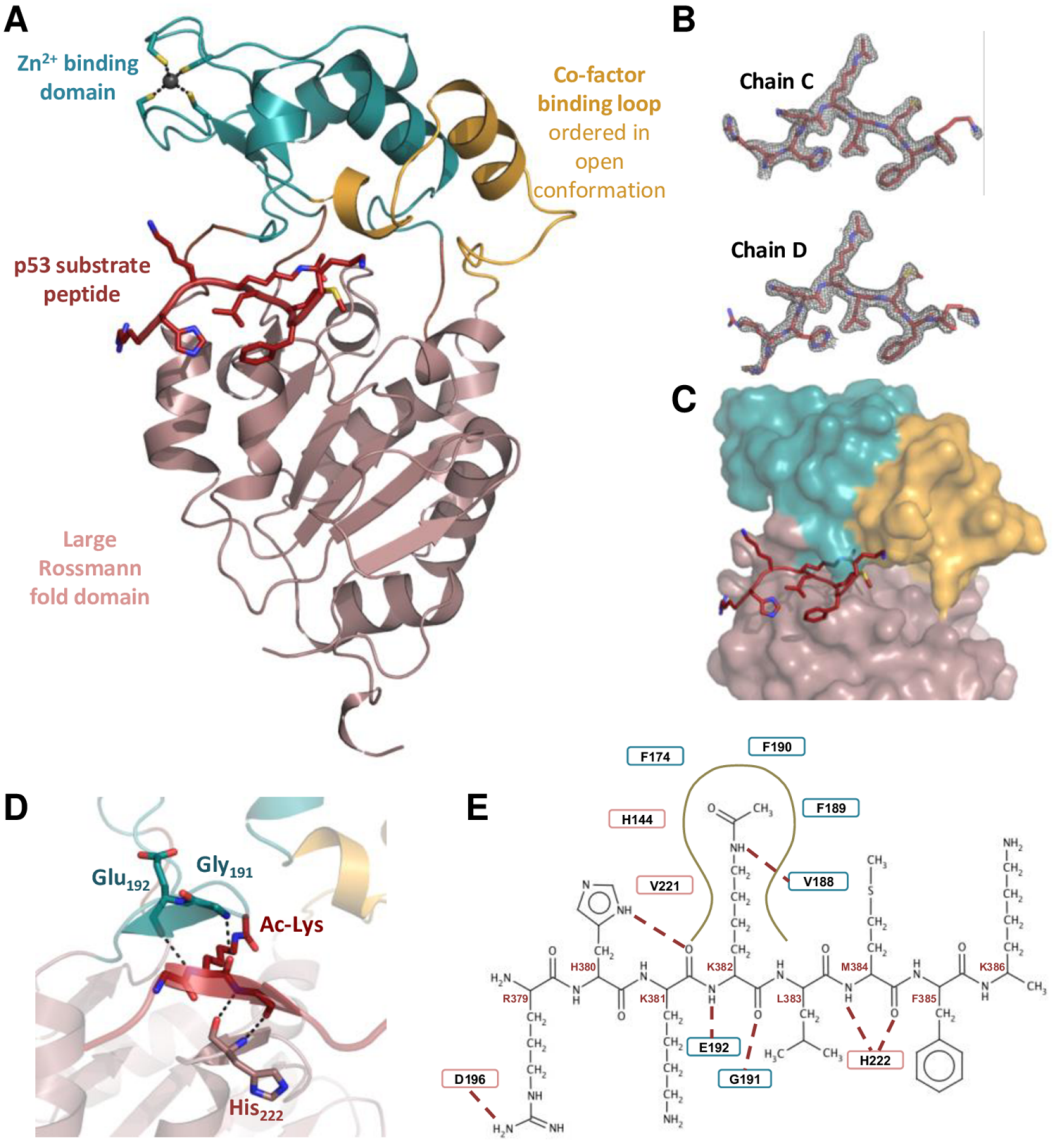
LiSIR2rp1(ΔP253-E303)/Acetylated p53 peptide complex structure. **(A)** Structure of LiSIR2rp1(ΔP253-E303) in complex with p53 peptide substrate. Large Rossmann-fold domain is colored in light pink, small domain in turquoise, cofactor binding loop in light orange and p53 peptide is depicted as sticks in red. **(B)** Electron densities (2Fo-Fc map contoured at 1σ) observed for acetylated p53 peptides (chains C and D) in both complexes of the asymmetric unit. Peptides are oriented as in D. **(C)** Surface representation of the LiSIR2rp1 in complex with p53 peptide (represented as sticks) binding to the cleft between the small and large domains. **(D)** Closer view of the interactions between LiSIR2rp1 and p53 peptide showing the three-stranded LiSIR2rp1-p53 β-sheet termed the β-staple (central substrate peptide strand surrounded on each side by β-strands contributed by both LiSIR2rp1 domains–Large Rossmann-fold and small zinc-binding domains) **(E)** Schematic representation of the interactions between LiSIR2rp1 and p53 peptide (residues numbered in red). Residues (single letter symbol with number) involved in main interactions through main chain (full line boxes) or side chain (dash line boxes) are represented. The red dashed lines indicate hydrogen bonds. Brown curve represents hydrophobic tunnel in which acetylated lysine inserts with surrounding residues implicated in hydrophobic and or van der Waals interactions with the acetyl-lysine. Residues belonging to the large Rossmann-fold domain and to the small domain are in pink and blue boxes, respectively.

The two protein monomers of the asymmetric unit are highly similar (rmsd of 0.4Å for all Cα atoms) with a typical sirtuin two-domain structure (Fig 3A)—a large Rossman-fold domain (pink) and a smaller zinc-binding domain (turquoise). The large domain is composed of six parallel β-strands, sandwiched between two layers of α-helices. The zinc-binding domain comprises a two-stranded antiparallel β-sheet with four α-helices and includes a Zn^2+^ ion coordinated by four strictly conserved cysteines (Cys152, 155, 176 and 179). The interface of the two domains forms a large groove, which accommodates the substrate peptide chain (Fig 3A–3C).

Structure-mapping of the tryptic digestion results (S3 Fig) shows that the 29kDa proteolytically stable fragment encompasses the small zinc-binding domain and a major part of the Rossmann-fold domain whilst the 9kDa domain comprises the remaining part of this large domain (two α-helices and one β-strand), with extensive interactions between both domains. This explains LiSIR2rp1 stability after tryptic digestion and the impossibility to produce the two proteolytically stable domains as independent recombinant polypeptides.

With similarity to that observed with human and yeast SIR2 homologues [8], structural comparisons of LiSIR2rp1(ΔP253-E303) complexed with p53 peptide with apo ScHst2, shows a rigid rotation of the *Leishmania* small zinc-binding domain towards the large domain (≈10°), closing the LiSIR2rp1 binding site (S4 Fig). Additionally, the cofactor-binding loop (amino acids 49–63) is ordered in the open conformation as described for ScHst2 in complex with p53 peptide substrate and carba-NAD (see S4 Fig) [51].

The p53 peptide binds into the cleft between the small and the large domain (Fig 3C). Eight and nine residues are visible among the 18-mer p53 peptide (R379-K386 and S378-K386 for chain C and D, respectively). The p53 peptide backbone forms an anti-parallel β-sheet like interaction termed the β-staple [8, 55], with main chain residues 191 and 192 of the small domain and residue 222 of the large domain (Fig 3D and 3E). The acetylated side chain of lysine 382 inserts into a hydrophobic tunnel delineated by Val 188 and Val 221. Deeper, the Nζ of the acetylated lysine forms a hydrogen bond with the carbonyl group of Val 188 main chain, and the acetyl group is trapped between Phe 74, His 144, Phe 189 and Phe 190. Additionally, the guanidinium group of Arg 379 is involved in hydrogen bonding with the Asp 196 side chain carboxyl group (Fig 3E).

### Superposition with human homologues reveals potential differences for the design of specific anti-LiSIR2rp1 compounds

**Fig 4:**
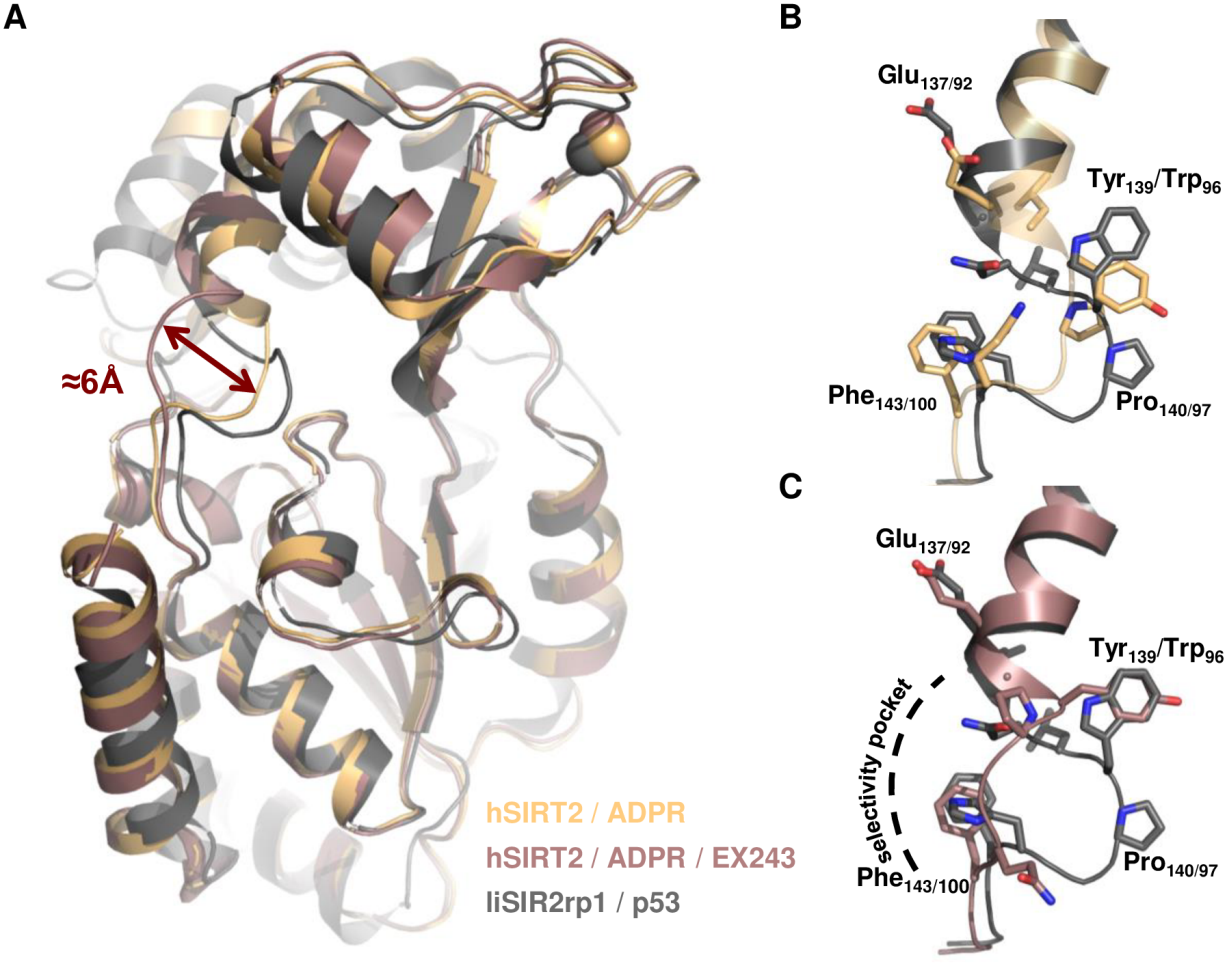
Selectivity pocket found in hSIRT2. **(A)** Superposition of LiSIR2rp1(ΔP253-E303)/Acetylated p53 peptide complex (grey) with hSIRT2 in complex with ADPR alone (pdb: 5d7o)(orange) or with EX243 inhibitor (pdb: 5d7p) (pink)(rmsd = 1.2Å). Upon binding of EX243, hSIRT2 active site rearranges and a new selectivity pocket forms. The hinge loop (residues 136–144) shifts of about 6Å. **(B)** and **(C)** Closer views of the hinge region of the structures shown in **(A)**. Residues of the hinge loop are depicted as sticks (first and second number refers to hSIRT2 and LiSIR2rp1, respectively).

Superposition of the LiSIR2rp1(ΔP253-E303)/p53 structure with sirtuin human homologue structures (hSIRT1-3) reveals a high degree of structural similarity (Fig 4A). The principal structural differences are encountered in the flexible cofactor-binding loop, which can adopt various conformations according to the sirtuins structures—i.e. unbound or in complex with different cofactors, substrates or small compounds [46]. The lowest rmsd values were calculated when LiSIR2rp1/p53 structure was superposed with human structures including ADP-ribose (ADPR) and peptide or pseudo-peptide substrates. Comparison with human homologue structures in complex with NAD, nicotinamide or some known inhibitors shows that most of the residues that form the NAD+ binding site (divided in three distinct pockets named A, B and C, respectively) [46] are conserved—as observed with residues interacting with the peptide.

In terms of isoform specificity, none of the residues identified as hot-spots by Parenti *et al*. [56] diverge between LiSIR2rp1 and the three closest human homologues SIRT1-3 in the NAD+ binding site (pockets A, B and C). However, selectivity for LiSIR2rp1 over human sirtuin could be considered by exploring the selectivity pocket described for hSIRT2 in several co-structures (hSIRT2 in complex with SIRT2 selective Sirtuin-rearranging ligand termed SirReal1 and 2 [57], EX243 and CHIC 35 [58] or inhibitor 29c [59]. In these structures, an additional pocket could be formed in the vicinity of the extended C-site (ECS) resulting from an active site rearrangement induced by the compound (Fig 4A). The residues delineating this region and implicated in compound interactions are loop 136–144 and 188–191 as well as the amino acids Thr171, Leu206 and Ile213 (following hSIRT2 numbering) [46]. Interestingly, the equivalent to hSIRT2 loop 136–144 in LiSIR2rp1 (residues 91–101) is extended due to the presence of two additional amino acids: Asn 94 and Leu 95 which correspond to a gap between Leu138 and Tyr139 in hSIRT2 (*i*.*e*. 136-KEL—YPGQFK-144 in hSIRT2 and 91-RELNLWPHFQ-101 in LiSIR2rp1) (Figs 2 and 4B). Furthermore, if most residues of this region, including the selectivity pocket are conserved, Tyr 139, Thr 171, Leu 206 and Ile 213 in hSIRT2 are substituted by Trp 96, Gly 128, Ile 161 and Ala 168 respectively in LiSIR2rp1 (Figs 2 and 4C). Together, these differences may highlight a potential for drug interactions that can differentiate between the parasite and human sirtuins, a key focus in *Leishmania* sp. selective drug targeting.

### Large serine-rich insertions (SRIs) in the β8-β9 connector of the Rossmann-fold are a distinctive feature of kinetoplastid SIR2rp1 proteins

**Fig 5:**
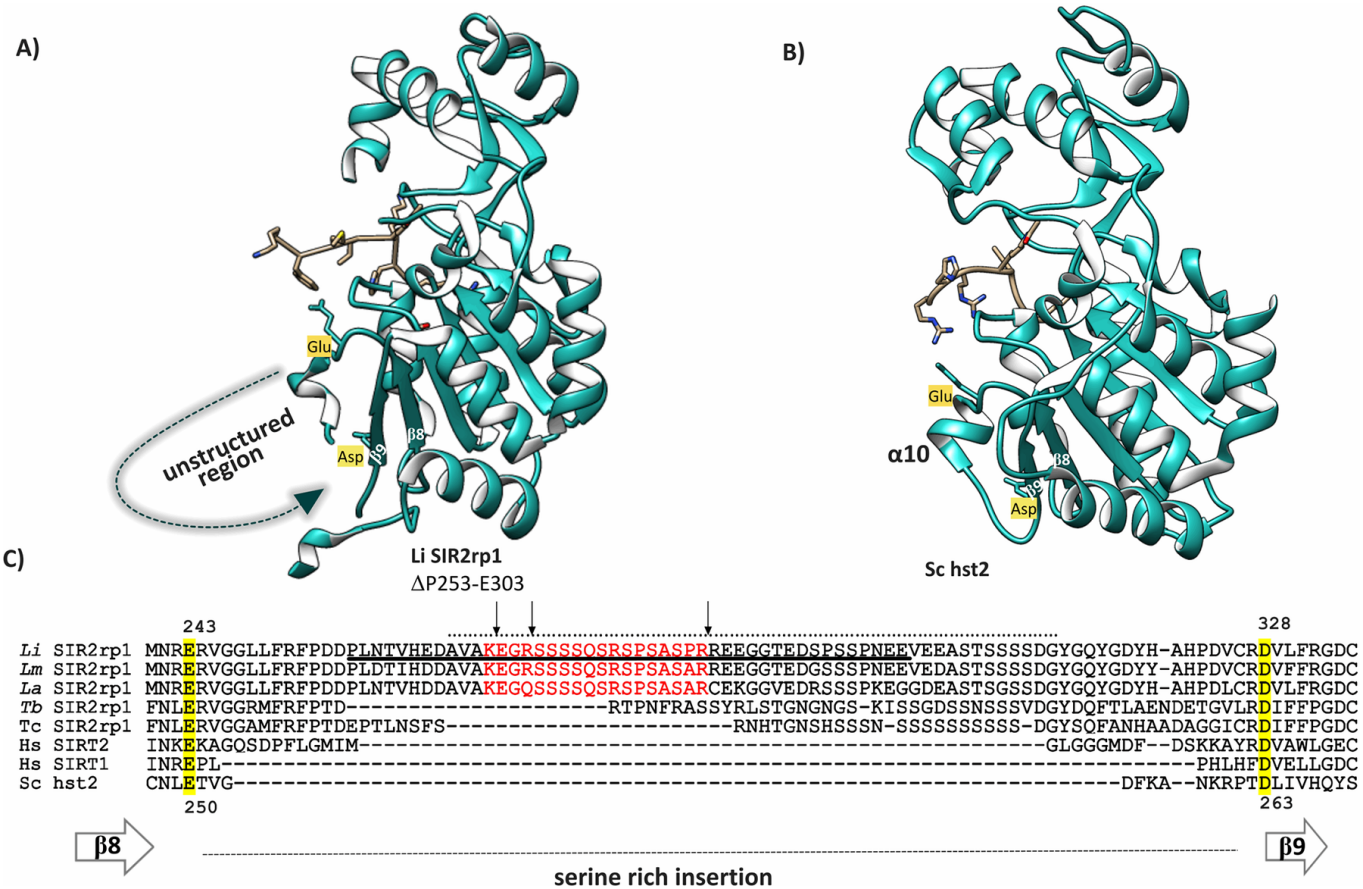
Extensive serine rich insertions in the β8-β9 connector region of the sirtuin Rossmann-fold domain is an exclusive feature of kinetoplastid parasite SIR2rp1 proteins. Multiple sequence alignment with SIRT2/Hst2/Sir2rp1 homologues indicates that the serine-rich disordered region, located between flanking β8 and β9 strands, is found only in kinetoplastid/trypanosomatid parasites. Structures of **(A)** the LiSIR2rp1 ΔP253-E303 mutant and **(B)** the yeast homolog ScHst2 (pdb: 1szc), focused on the α10 helical region with flanking β8 and β9 strands. The conserved Glu and Asp residues are appended to define the limits of the intervening peptide insertions. **(C)** Multiple sequence alignments with trypanosomatid SIR2rp1 proteins -versus human and yeast homologs, hSIRT1, hSIRT2 and ScHst2 (refer to S1 Table for more details). Numbering refers to conserved residues in LiSIR2rp1 (243–328) or *Sc*Hst2 (250–263). The proteolytically-sensitive region in *Leishmania spp*. is shown in red with arrows indicating trypsin cleavage sites. The region removed in LiSIR2rp1 ΔP253-E303 is underlined. Residues in LiSIR2rp1 predicted as disordered by DisEMBL (Remark 465 algorithm–S3 Text) are shown by the upper dotted line.

The occurrence of the large serine-rich β8-β9 connector insertion in LiSIR2rp1 appears from multiple sequence alignments to be specific to kinetoplastids, as evidenced from comparisons with hSIRT2 and ScHst2 homologues [8] and the primary sequences of multiple SIR2 homologues from a wide variety of organisms (Fig 2 and S1 Table). Removal of 51 amino acids of this loop in LiSIR2rp1 ΔP253-E303 did not impair proper folding of the crystallised protein, where flexibility in the missing residues 304–323 suggests the absence of artificial structural constraints upon deleting this region (Fig 5).

In ScHst2, the α10 helix region is bordered by the residues Glu250 and Asp263 which are highly conserved in multiple sequence alignments and define the limits of the flanking β strands (β8 and β9 shown in Fig 5A and 5B). The primary sequences occurring between these two reference points were compared for SIR2 proteins from different organisms (refer to S1 Table). Like *Sc*Hst2, hSIRT1-3 and *Sc*SIR2 only display narrow intervening sequence lengths ranging from 7 (hSIRT1, *Sc*Hst2) to 28 (hSIRT2) residues; none were serine-rich, acidic (low pI) or approach the length of the primary sequences that are characteristic of *Leishmania* spp. SIR2rp1 insertions (Fig 5C).

Large SRIs in the β8-β9 connector region were found exclusively in the SIR2rp1 proteins of the Trypanosomatida order of kinetoplastid parasites (although a general lack of protein sequence data precludes excluding alternative kinetoplastid orders). The most extensive expansions encompassing the proteolytically sensitive region appear to have occurred in the leishmaniinae, including *Leishmania* spp. and *Leptomonas* spp., and *Phytomonas* spp., where T*rypanosoma* spp. show distinctly smaller insertions with generally higher theoretical isoelectric points (e.g. 7.4–9.5 versus 3.9–4.8, see S1 Table). SRIs were found in the β8-β9 connector region of all kinetoplastid SIR2rp1 sequences analysed. However, they are absent from the SIR2rp2 and SIR2rp3 paralogues, which also show considerable sequence and functional divergence from SIR2rp1, belonging to different phylogenic clades associated with alternative cellular functions [20].

No large β8-β9 insertions have been detected in SIRT2 proteins of other protozoan parasites, including *Entamoeba histolytica*, the causative agent of amoebiasis, *Toxoplasma gondii*, (toxoplasmosis) and the malarial agent *Plasmodium falciparum*. Similarly, the SIRT2 proteins of the trematode parasites including *Schistosoma mansoni*, appear to possess relatively short β8-β9 connecting regions approximately similar to hSIRT2 (36 versus 28 residues respectively).

As well as direct experimental evidence from native tryptic digests and *in silico* modelling (above), computational disorder analysis of the SRI in LiSIR2rp1 strongly suggests that this region exists as intrinsically disordered region (IDR). Thus, residues D263 –G312, most of which are deleted from the β8-β9 connector in the crystallised LiSIR2rp1 ΔP253-E303 mutant, are defined as intrinsically disordered, with maximal probability (0.9) occurring in the vicinity of the trypsin sensitive linker region defined above (Fig 1 and DisEMBL, Remark 465 algorithm—see S3 Text) [39]. As with general trypanosomatid IDPs, this region is enriched in polar and charged residues (S, T, Q, R, D, E) as well as proline residues that oppose the establishment of stable secondary structures, whilst it possesses low amounts of hydrophobic and aromatic residues that promote order in the hydrophobic cores of stable globular proteins [60]. Genome-wide analysis in trypanosomatids has associated IDPs with functions enriched for binding and catalysis as well as pathways that include an inclination towards cell signaling functions and host-parasite interactions, such as invasion and survival within the host [60]. Whilst little is known of IDR functions in sirtuins, there is a strong suggestion that they promote intermolecular interactions due to the favorable thermodynamic consequences of coupled folding/binding, either directly or after targeted modification by alternative cell signalling enzymes [9, 61]. Indeed, primary sequence analysis of the LiSIR2rp1 SRI shows a strong potential for post-translational modification. By analogy with the importance of local unwinding on trypsin accessibility for rapid proteolysis, the intrinsically disordered nature of the SRI may accelerate interaction with parasite serine kinases and/or other post-translational modification (PTM) enzymes. Indeed, the conformational requirements for PTMs by several different enzymes have been proposed to be promoted by structural disorder [61], none more widely studied than the diverse covalent modifications targeting the disordered tails of histones [62]. A strong correlation has also been observed between annotated PTMs and intrinsic disorder in protein structures solved by NMR [63]. Significantly, the molecular characteristics of protein regions surrounding protein phosphorylation sites share distinct similarities with IDRs [64]. In this respect, 14 different potential serine phosphorylation sites are located in the intrinsically disordered region D263 –G312 of LiSIR2rp1 (KinasePhos 2.0) [65]. Furthermore, of the 31 serine residues in LiSIR2rp1, all 13 potential serine phosphorylation sites detected by the DISorder-enhanced PHOSphorylation predictor occur in the SRI of the β8-β9 connector (S4 Text) [64]. Moreover, 8 of these serine residues reside within the disordered linker region defined above (colored red in Figs 1A and 5B) are shown experimentally to be highly accessible to trypsin, and by extrapolation readily available to protein kinases and/or other PTM enzymes.

### Assessing the biological role of the disordered *Li*SIR2rp1 β8-β9 connector in *L*. *infantum* promastigotes

**Fig 6:**
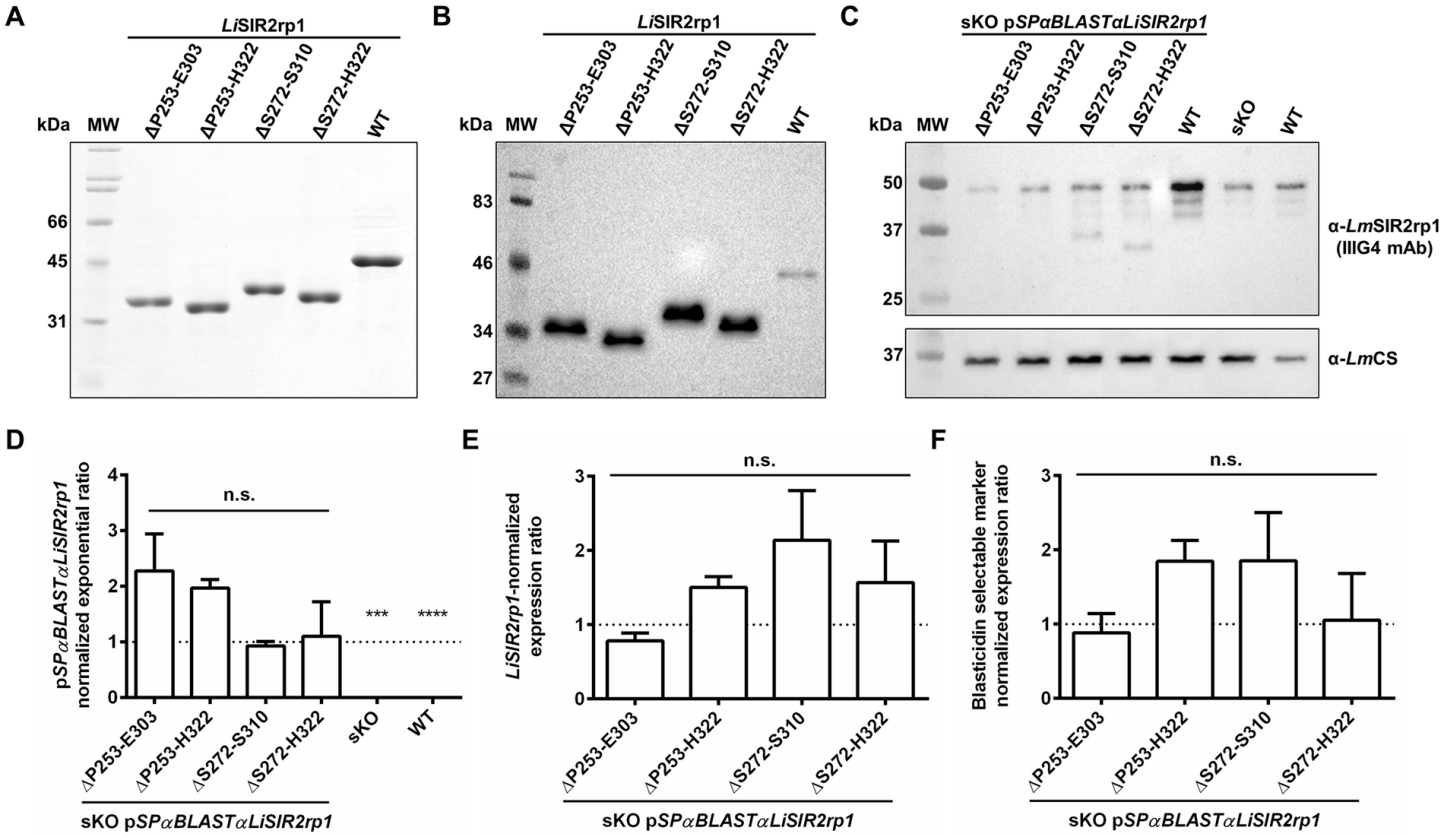
Expression of LiSIR2rp1 truncated forms in promastigotes. **(A)** Coomassie blue-stained SDS-PAGE gel of 5 μg of recombinant LiSIR2rp1 protein (WT) and respective truncated forms (ΔP253-E303; ΔP253-H322; ΔS272-S310; ΔS272-H322). **(B)** Western blot showing recognition of all LiSIR2rp1 recombinant forms by anti-L. major SIR2rp1 (α-LmSIR2rp1) IIIG4 monoclonal antibody (mAb). **(C)** Western blot analysis of LiSIR2rp1 levels in LiSIR2rp1 single knockout (sKO) promastigotes complemented with each pSPαBLASTα*Li*SIR2rp1 construct, as well as in sKO and WT parasites. Cysteine synthase (CS) was used as the loading control. **(D)** Quantification of pSPαBLASTα*Li*SIR2rp1 constructs by qPCR using genomic DNA of transfected, sKO and WT parasites, relative to sKO pSPαBLASTα*Li*SIR2rp1^WT^. **(E, F)** Expression levels of LiSIR2rp1 **(E)** and the blasticidin resistance marker **(F)** determined by qPCR using cDNA from transfected parasites, relative to sKO pSPαBLASTα*Li*SIR2rp1^WT^. Real time quantitative PCR (qPCR) analysis was performed using *LirRNA45* as the reference gene. Means and standard deviations of two independent experiments are represented. Unpaired t-test with Welch’s correction was performed: ***, p < 0.01; ****, p < 0.0001; n.s., non-significant.

To investigate the biological role of LiSIR2RP1 disordered β8-β9 connector region, the sequences encoding for either the entire protein or the deletion mutants (ΔP253-E303; ΔP253-H322; ΔS272-S310; ΔS272-H322) were sub-cloned into the pSP72αBLASTα episomal vector. These constructs were further used to transfect LiSIR2rp1 single knockout promastigotes aiming to evaluate whether the expression of the mutant forms could, as the full-length LiSIR2rp1, rescue the defective phenotype of parasites in the amastigote stage [22]. We first analysed whether promastigotes are able to express the truncated forms of LiSIR2rp1. For that, we evaluated whether the recombinant LiSIR2rp1 deletion mutants (Fig 6A) are recognized by the anti-LmSIR2rp1 monoclonal antibody (IIIG4) (Fig 6B) [40] and then investigated the LiSIR2rp1 expression profile on transfectant extracts (Fig 6C). As previously reported [22], single knockout parasites show lower levels of LiSIR2rp1 compared to WT (35–67% of WT in 4 independent experiments). Promastigotes carrying the episomal copy of the WT LiSIR2rp1 function as an overexpressing line showing increased levels of LiSIR2rp1 compared to WT promastigotes. However, mutant LiSIR2rp1 proteins appear to not be expressed as effectively (Fig 6C). In fact, ΔP253-E303 and ΔP253-H322 LiSIR2rp1 remained undetectable by Western blot in the transfectant extracts under the conditions tested. In an attempt to shed light onto the reason for the defective expression of the mutated forms, plasmid copy numbers and mRNA levels of both LiSIR2rp1 and the selectable marker were determined by quantitative PCR (Fig 6D–6F). No statistically significant differences between the different transfectants were found, revealing an unimpaired transcription of the various episomal forms of LiSIR2rp1. Overall, these data suggest that the extended LiSIR2rp1 β8-β9 connector region may be required for protein translation and/or stability in parasites. At the present time, the lack or lower expression of the deleted forms in the single knockout parasites compared to the full length LiSIR2rp1, has precluded further studies particularly to assess the eventual rescue of the amastigotes defective phenotype (see Discussion).

## Discussion

Deleting 51 residues from a proteolytically-sensitive region connecting the β8 and β9 strands of the catalytic core led to the crystallization and structural resolution of *Leishmania infantum* SIR2rp1. This approach has allowed the first direct insight into the molecular structure of a sirtuin belonging to a kinetoplastid parasite, the protozoan agents responsible for considerable burden of disease worldwide.

In LiSIR2rp1, the usual β8-α10-β9-fold embodied in hSIRT2 and ScHst2 homologues is replaced by a large region, predicted theoretically and experimentally to be intrinsically disordered, connecting parallel β8 and β9 strands of the six stranded β-sheet. This unusually large SRI in the β8-β9 connector of the Rossmann-fold appears to be a molecular signature unique to kinetoplastid parasite Sir2rp1 proteins, where its function remains unknown. The loop spanning 293–307 of the smaller β8-β9 connector insertion in hSIRT2 has previously been noted to act as a substrate peptide mimetic that adopts alternative conformations dependent on substrate and NAD^+^ binding [47]. No evidence for such interactions was observed in the present LiSIR2rp1 structure.

The presence of the β8-β9 connector in LiSIR2rp1 is neither required for NAD^+^-dependent deacetylase activity, nor does it appear to confer a direct regulatory role on the deacetylase activity of the catalytic core. Removal of large parts of the LiSIR2rp1 β8-β9 connector loop in targeted deletion mutants did not alter substrate or cofactor binding properties substantially in deacetylation assays. Whilst deletion mutant proteins (ΔL257-S282 and ΔP253-E303) appear to show moderately lower specific activities (and kcat values) when compared to the full-length protein (Table 1), these observations do not apply to LiSIR2rp1 when portions of the intervening loop were removed directly by proteolysis (Fig 1). Since LiSIR2rp1 deletion mutants produced similar yields when expressed in *E*. *coli*, the reason for their lower stability and/or accumulation when expressed in parasite cells remains to be understood. Removal of the IDR along with its strong potential for post-translational modification may reduce stability, folding, protein binding and/or compartmentalization in the parasite cell. In this latter respect, the careful design of future deletion mutants to possess suitable replacements of the β8-β9 connector loop engineered to maintain structural integrity and stability in parasites would greatly facilitate studies of the biological function of this unique addition to kinetoplastid sirtuins.

In addition to a central catalytic core, sirtuins are commonly flanked by extensions to their N- and C-termini. The extensions vary in sequence and length, and can impart distinctive functions including oligomerisation and auto-regulation (reviewed in [8]) as well as a potential for IDRs and post-translational modification sites present in the protein termini to direct other sirtuin specific cellular functions [9, 60, 66]. In the latter respect, the most extensive N and C-terminal additions, as seen in hSIRT1, are mostly absent from LiSIR2RP1, which locates to the cytoplasm [21] showing similarity to hSIRT2 [6], rather than the nucleus as with hSIRT1 [67]. The lack of a direct regulatory function of the LiSIR2rp1 β8-β9 connector loop on catalytic efficiency is also contrary to the functions of the N- and C-terminal extensions of hSIRT1, which stimulate deacetylase activity intra- and inter-molecularly [13, 68]. Nonetheless, we note that the amino acid composition of the 84 residue β8-β9 connector region within the Rossmann-fold of LiSIR2rp1 shows notable compositional similarities with the C-terminal 90 residues of hSIRT1; both are acidic (isoelectric points ≤ 4.4), serine rich (ser ≥ 17%) and essentially predicted as intrinsically disordered and largely removed in order to obtain the corresponding crystal structures [13]. Also, the composition of LiSIR2rp1 β8-β9 connector and C-terminus of hSIRT1 both show high potential for post translational modification, notably by phosphorylation or glycosylation [66, 69]. Whilst little is known of the impact of such modifications on the regulation and/or cell targeting of the respective sirtuins, it is curious to speculate that such modifications may modulate LiSIR2rp1 in a fashion potentially mimicking, as yet unknown, hSIRT1 C-terminal functions. The SIR2rp1 β8-β9 connector thereby appears to offer an alternative internal location (neither C- nor N-terminal as is more usual for this family of proteins), situated in the proximity of the NAD^+^-binding domain, to provide as yet uncharacterised accessory functions to the corresponding kinetoplastid sirtuins. Whilst the short β8-β9 connector acts as a peptide mimetic in hSIRT2 crystal dimers, the even shorter β8-β9 connector loop of hSIRT1 is known to provide important intramolecular interactions with the C-terminal regulatory (CTR) segment that stabilize the catalytic core of hSIRT1 [13]. Additional molecular functions may be anticipated for the larger, unstructured β8-β9 connector in kinetoplastid parasites.

The principal structural elements of the catalytic core, responsible for substrate and cofactor binding as well as catalysis, are remarkably conserved between sirtuins across all kingdoms of life [8]. The LiSIR2rp1 core displays a high degree of structural superposition with other sirtuins from the same phylogenic subclass 1, including those of the host, hSIRT1-3 [20]. Substrate binding involves several non-selective hydrogen bonding interactions from LiSIR2rp1 with main chain atoms along the peptide backbone, where the acetyl-lysine moiety is accommodated in a hydrophobic tunnel via several van der Waals interactions with mostly aromatic hydrophobic residues. The limited substrate sequence selectivity observed in the LiSIRrp1/p53 structure is in keeping with catalytic core structures of other sirtuin homologues bound to their acetyl-lysine peptide substrates. Rather than primary sequence, selectivity is observed in a global protein context to the ability of the target substrate to adopt the required β-staple form upon binding to the catalytic site [55].

Despite their structural similarity and the paucity of sequence specific binding features, small differences in the respective catalytic sites of the corresponding host and parasite sirtuin proteins may now be explored for selective binding using structure-based design with co-crystallized inhibitors. A fragment based approach that previously led to the discovery of potent inhibitors of hSIRT2 with >200-fold and > 900-fold selectivity over hSIRT1 and hSIRT3 respectively confirms that the highly conserved core sirtuin functions do possess sufficient variances at a structural level to enable specific targeting [70].

Conservation of several residues delineating the selectivity pocket observed in hSIRT2 suggests that LiSIR2rp1 could accommodate SirReal-like molecules [57] while sequence specific differences and the presence of two additional amino acids in LiSIR2rp1 may add some flexibility and/or specificity to the ECS and selectivity pocket (Fig 4). Further analyses focused on these observations and supported by the *de novo* structural information will facilitate the search for new LiSIR2rp1 inhibitors. As well as gaining novel mechanistic insights into sirtuin functions in a kinetoplastid parasite, the present report represents an important first step towards the structure-based design of selective compounds against this essential *Leishmania* protein.

## Supporting information

S1 Fig Trypsin hyper-sensitivity of the internal serine-rich region linking stable N and C-terminal regions provides experimental evidence for an intrinsically disordered region. Native trypsin digestion of the hexa histidine tagged LiSIR2rp1 protein was carried out as described for Fig 1 (see Methods). **(A)** Time resolved native digests showing (i) the rapid loss of full length (42 kDa) protein with concomitant appearance of a His tagged 31 kDa fragment (at 2s) and (ii) the subsequent removal of the N-terminal His tag and the first six LiSIR2rp1 residues from the 31 kDa fragment to yield a stable 29 kDa fragment (at 90s), as described in the text. The presence or absence of NAD^+^ had no effect on the digestion pattern. **(B)** and **(D)** Time resolved digestion and SDS PAGE analysis of the 31kDa to 29 kDa proteolysis (cleavage at R6) and associated quantification of relative band intensities over time. **(C)** and **(E)** Time resolved digestion and SDS PAGE analysis of the proteolytic disappearance of the 29 kDa N-terminal fragment and associated quantification of relative band intensity over time. **(F)** Schematic representation of the data (A-E), showing the principal proteolytic cleavage sites, the corresponding N-terminal fragments, and comparison of their relative half-lives (in seconds). Green bars represent portions of the protein predicted to be disordered (DisEMBL, Remark 465 algorithm) (Linding et al, 2003). Quantitative data was fitted to first order exponential decay [y = A_0_.exp^(-kt)^] or association [y = A_0_ (1- exp^(-kt)^)] models where A_0_ = 100% (GraphPad prism 6.0 software).
(TIF)

S2 Fig LiSIR2rp1 human (A) and yeast (B) homologue based models. Models (i) and (ii) are based on hSIRT2 pdb 3zgo and model (iii) on hSIRT2 pdb 1j8f. Models (iv), (v) and (vi) are based on ScHst2 pdb 1q14, 1q17 and 1q1a, respectively. The large Rossmann-fold and small zinc-binding domains are colored in turquoise and pink respectively. The LiSIR2RP1 insertion region is colored in red (amino acids 250–300 and 250–320 for human- and yeast-homologue based model respectively).
(TIF)

S3 Fig Structure-mapping of the stable fragments of native tryptic digests. The two proteolytically-stable domains are colored in the same way as Fig 1. The p53 peptide substrate is in grey with acetyl-lysine side chain depicted as sticks.
(TIF)

S4 Fig Superposition of LiSIR2rp1(ΔP253-E303)/p53 with various ScHst2 complex structures indicate conformational changes of LiSIR2rp1 upon substrate binding. LiSIR2rp1(ΔP253-E303)/p53 is colored as in Fig 3 while ScHst2 is shown in grey. The acetyl-lysine residue, substrate analogue (carba-NAD) and product (2’-O-acetyl-ADP-ribose) are shown as sticks. **A**. Superposition of LiSIR2rp1(ΔP253-E303)/p53 complex with apo ScHst2 (pdb: 1q14) evidenced a rigid rotation of Leishmania small zinc-binding domain towards the large Rossmann-fold domain (≈10°). The ScHst2 cofactor-binding loop is disordered (dashed line). (ScHst2 C-ter α-helix 13 was removed for clarity). **B**. The LiSIR2rp1 co-factor binding loop adopts the ordered open conformation as described for ScHst2 in presence of peptide substrate and carba-NAD (pdb: 1zsc). **C**. When bound to peptide substrate and product, the ScHst2 cofactor binding loop is in a closed conformation.
(TIF)

S1 Report Validation report for crystal structure PDB reference 5Ol0 from worldwide protein data bank. (PDF)

S1 Table Comparison of the β8—β9 connector of the Rossmann-fold domain of SIR2 (ScHst2/hSIRT2) identifies a serine-rich peptide extension unique to SIR2rp1 proteins of kinetoplastid/trypanosomatid parasites. (PDF)

S2 Table Oligonucleotide sequences used in the generation of deletion constructs for crystallography (primers 1 and 2 coupled with the corresponding 2 primers for each deletion mutant). (PDF)

S3 Table Oligonucleotide sequences used in the generation of constructs and quantitative PCR. (PDF)

S1 Text Analysis of native tryptic digest of LiSIR2rp1 by LC/ ESI-TOF MS. (PDF)

S2 Text Design of LiSIR2rp1 deleted mutants for structural studies. (PDF)

S3 Text Intrinsic disorder prediction for LiSIR2rp1 using the DisEmble Remark 465 algorithm. (PDF)

S4 Text Phosphorylation site prediction in LiSIR2rp1 using the DISorder-enhanced PHOSphorylation predictor detects 13 potential sites all within the β8—β9 connector region. (PDF)

## References

[1] WHO. Investing to overcome the global impact of neglected tropical diseases. Third WHO report on neglected tropical diseases. 2015;90:33–43.

[2] Drazic A, Myklebust LM, Ree R, Arnesen T. The world of protein acetylation. Biochim Biophys Acta. 2016;1864(10):1372–401. Epub 2016/06/15. doi: 10.1016/j.bbapap.2016.06.007 . 27296530 10.1016/j.bbapap.2016.06.007

[3] Seto E, Yoshida M. Erasers of histone acetylation: the histone deacetylase enzymes. Cold Spring Harb Perspect Biol. 2014;6(4):a018713 Epub 2014/04/03. doi: 10.1101/cshperspect.a018713 . 24691964 10.1101/cshperspect.a018713 PMC3970420

[4] Verdin E, Ott M. 50 years of protein acetylation: from gene regulation to epigenetics, metabolism and beyond. Nat Rev Mol Cell Biol. 2015;16(4):258–64. Epub 2015/01/01. doi: 10.1038/nrm3931 . 25549891 10.1038/nrm3931

[5] Imai S, Armstrong CM, Kaeberlein M, Guarente L. Transcriptional silencing and longevity protein Sir2 is an NAD-dependent histone deacetylase. Nature. 2000;403(6771):795–800. Epub 2000/02/29. doi: 10.1038/35001622 . 10693811 10.1038/35001622

[6] North BJ, Marshall BL, Borra MT, Denu JM, Verdin E. The human Sir2 ortholog, SIRT2, is an NAD+-dependent tubulin deacetylase. Mol Cell. 2003;11(2):437–44. Epub 2003/03/07. . 12620231 10.1016/s1097-2765(03)00038-8

[7] Starai VJ, Celic I, Cole RN, Boeke JD, Escalante-Semerena JC. Sir2-dependent activation of acetyl-CoA synthetase by deacetylation of active lysine. Science. 2002;298(5602):2390–2. Epub 2002/12/21. doi: 10.1126/science.1077650 . 12493915 10.1126/science.1077650

[8] Sanders BD, Jackson B, Marmorstein R. Structural basis for sirtuin function: what we know and what we don’t. Biochim Biophys Acta. 2010;1804(8):1604–16. Epub 2009/09/22. doi: 10.1016/j.bbapap.2009.09.009 . 19766737 10.1016/j.bbapap.2009.09.009 PMC2886166

[9] Costantini S, Sharma A, Raucci R, Costantini M, Autiero I, Colonna G. Genealogy of an ancient protein family: the Sirtuins, a family of disordered members. BMC Evol Biol. 2013;13:60 Epub 2013/03/19. doi: 10.1186/1471-2148-13-60 . 23497088 10.1186/1471-2148-13-60 PMC3599600

[10] Khan AN, Lewis PN. Unstructured conformations are a substrate requirement for the Sir2 family of NAD-dependent protein deacetylases. J Biol Chem. 2005;280(43):36073–8. Epub 2005/09/01. doi: 10.1074/jbc.M508247200 . 16131486 10.1074/jbc.M508247200

[11] Hoff KG, Avalos JL, Sens K, Wolberger C. Insights into the sirtuin mechanism from ternary complexes containing NAD+ and acetylated peptide. Structure. 2006;14(8):1231–40. Epub 2006/08/15. doi: 10.1016/j.str.2006.06.006 . 16905097 10.1016/j.str.2006.06.006

[12] Cosgrove MS, Bever K, Avalos JL, Muhammad S, Zhang X, Wolberger C. The structural basis of sirtuin substrate affinity. Biochemistry. 2006;45(24):7511–21. Epub 2006/06/14. doi: 10.1021/bi0526332 . 16768447 10.1021/bi0526332

[13] Davenport AM, Huber FM, Hoelz A. Structural and functional analysis of human SIRT1. J Mol Biol. 2014;426(3):526–41. Epub 2013/10/15. doi: 10.1016/j.jmb.2013.10.009 . 24120939 10.1016/j.jmb.2013.10.009 PMC4211926

[14] Frye RA. Characterization of five human cDNAs with homology to the yeast SIR2 gene: Sir2-like proteins (sirtuins) metabolize NAD and may have protein ADP-ribosyltransferase activity. Biochem Biophys Res Commun. 1999;260(1):273–9. Epub 1999/06/25. doi: 10.1006/bbrc.1999.0897 . 10381378 10.1006/bbrc.1999.0897

[15] Zheng W. Sirtuins as emerging anti-parasitic targets. Eur J Med Chem. 2013;59:132–40. Epub 2012/12/12. doi: 10.1016/j.ejmech.2012.11.014 . 23220641 10.1016/j.ejmech.2012.11.014

[16] Tanny JC, Dowd GJ, Huang J, Hilz H, Moazed D. An enzymatic activity in the yeast Sir2 protein that is essential for gene silencing. Cell. 1999;99(7):735–45. Epub 2000/01/05. . 10619427 10.1016/s0092-8674(00)81671-2

[17] Garcia-Salcedo JA, Gijon P, Nolan DP, Tebabi P, Pays E. A chromosomal SIR2 homologue with both histone NAD-dependent ADP-ribosyltransferase and deacetylase activities is involved in DNA repair in Trypanosoma brucei. EMBO J. 2003;22(21):5851–62. Epub 2003/11/01. doi: 10.1093/emboj/cdg553 . 14592982 10.1093/emboj/cdg553 PMC275410

[18] da Mota FF, Marinho LP, Moreira CJ, Lima MM, Mello CB, Garcia ES, et al Cultivation-independent methods reveal differences among bacterial gut microbiota in triatomine vectors of Chagas disease. PLoS Negl Trop Dis. 2012;6(5):e1631 Epub 2012/05/09. doi: 10.1371/journal.pntd.0001631 . 22563511 10.1371/journal.pntd.0001631 PMC3341335

[19] Tavares J, Ouaissi M, Ouaissi A, Cordeiro-da-Silva A. Characterization of the anti-Leishmania effect induced by cisplatin, an anticancer drug. Acta Trop. 2007;103(2):133–41. Epub 2007/07/31. doi: 10.1016/j.actatropica.2007.05.017 . 17658446 10.1016/j.actatropica.2007.05.017

[20] Religa AA, Waters AP. Sirtuins of parasitic protozoa: in search of function(s). Mol Biochem Parasitol. 2012;185(2):71–88. Epub 2012/08/22. doi: 10.1016/j.molbiopara.2012.08.003 . 22906508 10.1016/j.molbiopara.2012.08.003 PMC3484402

[21] Tavares J, Ouaissi A, Santarem N, Sereno D, Vergnes B, Sampaio P, et al The Leishmania infantum cytosolic SIR2-related protein 1 (LiSIR2RP1) is an NAD+ -dependent deacetylase and ADP-ribosyltransferase. Biochem J. 2008;415(3):377–86. Epub 2008/07/05. doi: 10.1042/BJ20080666 . 18598238 10.1042/BJ20080666

[22] Vergnes B, Sereno D, Tavares J, Cordeiro-da-Silva A, Vanhille L, Madjidian-Sereno N, et al Targeted disruption of cytosolic SIR2 deacetylase discloses its essential role in Leishmania survival and proliferation. Gene. 2005;363:85–96. Epub 2005/10/21. doi: 10.1016/j.gene.2005.06.047 . 16236469 10.1016/j.gene.2005.06.047

[23] Moreira D, Santarem N, Loureiro I, Tavares J, Silva AM, Amorim AM, et al Impact of continuous axenic cultivation in Leishmania infantum virulence. PLoS Negl Trop Dis. 2012;6(1):e1469 Epub 2012/02/01. doi: 10.1371/journal.pntd.0001469 . 22292094 10.1371/journal.pntd.0001469 PMC3265455

[24] Linding R, Russell RB, Neduva V, Gibson TJ. GlobPlot: Exploring protein sequences for globularity and disorder. Nucleic Acids Res. 2003;31(13):3701–8. Epub 2003/06/26. . 12824398 10.1093/nar/gkg519 PMC169197

[25] Dosztanyi Z, Csizmok V, Tompa P, Simon I. IUPred: web server for the prediction of intrinsically unstructured regions of proteins based on estimated energy content. Bioinformatics. 2005;21(16):3433–4. Epub 2005/06/16. doi: 10.1093/bioinformatics/bti541 . 15955779 10.1093/bioinformatics/bti541

[26] Combet C, Blanchet C, Geourjon C, Deleage G. NPS@: network protein sequence analysis. Trends Biochem Sci. 2000;25(3):147–50. Epub 2000/03/01. . 10694887 10.1016/s0968-0004(99)01540-6

[27] McGuffin LJ, Bryson K, Jones DT. The PSIPRED protein structure prediction server. Bioinformatics. 2000;16(4):404–5. Epub 2000/06/27. . 10869041 10.1093/bioinformatics/16.4.404

[28] Larkin MA, Blackshields G, Brown NP, Chenna R, McGettigan PA, McWilliam H, et al Clustal W and Clustal X version 2.0. Bioinformatics. 2007;23(21):2947–8. Epub 2007/09/12. doi: 10.1093/bioinformatics/btm404 . 17846036 10.1093/bioinformatics/btm404

[29] Arnold K, Bordoli L, Kopp J, Schwede T. The SWISS-MODEL workspace: a web-based environment for protein structure homology modelling. Bioinformatics. 2006;22(2):195–201. Epub 2005/11/23. doi: 10.1093/bioinformatics/bti770 . 16301204 10.1093/bioinformatics/bti770

[30] Kabsch W. Xds. Acta Crystallogr D Biol Crystallogr. 2010;66(Pt 2):125–32. Epub 2010/02/04. doi: 10.1107/S0907444909047337 . 20124692 10.1107/S0907444909047337 PMC2815665

[31] Winn MD, Ballard CC, Cowtan KD, Dodson EJ, Emsley P, Evans PR, et al Overview of the CCP4 suite and current developments. Acta Crystallogr D Biol Crystallogr. 2011;67(Pt 4):235–42. Epub 2011/04/05. doi: 10.1107/S0907444910045749 . 21460441 10.1107/S0907444910045749 PMC3069738

[32] Keegan RM, Winn MD. Automated search-model discovery and preparation for structure solution by molecular replacement. Acta Crystallogr D Biol Crystallogr. 2007;63(Pt 4):447–57. Epub 2007/03/21. doi: 10.1107/S0907444907002661 . 17372348 10.1107/S0907444907002661

[33] Vagin A TA. MOLREP: an automated program for molecular replacement. J Appl Crystallogr. 1997;30(6):1022–5.

[34] Stein N. CHAINSAW: a program for mutating pdb files used as templates in molecular replacement. J Appl Crystallogr. 2008;41:641–3.

[35] Emsley P, Lohkamp B, Scott WG, Cowtan K. Features and development of Coot. Acta Crystallogr D Biol Crystallogr. 2010;66(Pt 4):486–501. Epub 2010/04/13. doi: 10.1107/S0907444910007493 . 20383002 10.1107/S0907444910007493 PMC2852313

[36] Murshudov GN, Skubak P, Lebedev AA, Pannu NS, Steiner RA, Nicholls RA, et al REFMAC5 for the refinement of macromolecular crystal structures. Acta Crystallogr D Biol Crystallogr. 2011;67(Pt 4):355–67. Epub 2011/04/05. doi: 10.1107/S0907444911001314 . 21460454 10.1107/S0907444911001314 PMC3069751

[37] DeLano W. The PyMOL Molecular Graphics System San Carlos, CA USA: DeLano Scientific; 2002 [25/01/2018]. https://pymol.org/2/.

[38] Pettersen EF, Goddard TD, Huang CC, Couch GS, Greenblatt DM, Meng EC, et al UCSF Chimera—a visualization system for exploratory research and analysis. J Comput Chem. 2004;25(13):1605–12. Epub 2004/07/21. doi: 10.1002/jcc.20084 . 15264254 10.1002/jcc.20084

[39] Linding R, Jensen LJ, Diella F, Bork P, Gibson TJ, Russell RB. Protein disorder prediction: implications for structural proteomics. Structure. 2003;11(11):1453–9. Epub 2003/11/08. . 14604535 10.1016/j.str.2003.10.002

[40] Vergnes B, Sereno D, Madjidian-Sereno N, Lemesre JL, Ouaissi A. Cytoplasmic SIR2 homologue overexpression promotes survival of Leishmania parasites by preventing programmed cell death. Gene. 2002;296(1–2):139–50. Epub 2002/10/18. . 12383511 10.1016/s0378-1119(02)00842-9

[41] Faria J, Loureiro I, Santarem N, Cecilio P, Macedo-Ribeiro S, Tavares J, et al Disclosing the essentiality of ribose-5-phosphate isomerase B in Trypanosomatids. Sci Rep. 2016;6:26937 Epub 2016/05/28. doi: 10.1038/srep26937 . 27230471 10.1038/srep26937 PMC4882579

[42] Pfaffl MW. A new mathematical model for relative quantification in real-time RT-PCR. Nucleic Acids Res. 2001;29(9):e45 Epub 2001/05/09. . 11328886 10.1093/nar/29.9.e45 PMC55695

[43] Johnson DE, Xue B, Sickmeier MD, Meng J, Cortese MS, Oldfield CJ, et al High-throughput characterization of intrinsic disorder in proteins from the Protein Structure Initiative. J Struct Biol. 2012;180(1):201–15. Epub 2012/06/02. doi: 10.1016/j.jsb.2012.05.013 . 22651963 10.1016/j.jsb.2012.05.013 PMC3578346

[44] Hubbard SJ, Eisenmenger F, Thornton JM. Modeling studies of the change in conformation required for cleavage of limited proteolytic sites. Protein Sci. 1994;3(5):757–68. Epub 1994/05/01. doi: 10.1002/pro.5560030505 . 7520312 10.1002/pro.5560030505 PMC2142727

[45] Hubbard SJ, Beynon RJ, Thornton JM. Assessment of conformational parameters as predictors of limited proteolytic sites in native protein structures. Protein Eng. 1998;11(5):349–59. Epub 1998/07/29. . 9681867 10.1093/protein/11.5.349

[46] Sacconnay L, Carrupt PA, Nurisso A. Human sirtuins: Structures and flexibility. J Struct Biol. 2016;196(3):534–42. Epub 2016/10/30. doi: 10.1016/j.jsb.2016.10.008 . 27773637 10.1016/j.jsb.2016.10.008

[47] Moniot S, Schutkowski M, Steegborn C. Crystal structure analysis of human Sirt2 and its ADP-ribose complex. J Struct Biol. 2013;182(2):136–43. Epub 2013/03/05. doi: 10.1016/j.jsb.2013.02.012 . 23454361 10.1016/j.jsb.2013.02.012

[48] Finnin MS, Donigian JR, Pavletich NP. Structure of the histone deacetylase SIRT2. Nat Struct Biol. 2001;8(7):621–5. Epub 2001/06/28. doi: 10.1038/89668 . 11427894 10.1038/89668

[49] Zhao K, Chai X, Clements A, Marmorstein R. Structure and autoregulation of the yeast Hst2 homolog of Sir2. Nat Struct Biol. 2003;10(10):864–71. Epub 2003/09/23. doi: 10.1038/nsb978 . 14502267 10.1038/nsb978

[50] Zhao K, Chai X, Marmorstein R. Structure of the yeast Hst2 protein deacetylase in ternary complex with 2'-O-acetyl ADP ribose and histone peptide. Structure. 2003;11(11):1403–11. Epub 2003/11/08. . 14604530 10.1016/j.str.2003.09.016

[51] Zhao K, Harshaw R, Chai X, Marmorstein R. Structural basis for nicotinamide cleavage and ADP-ribose transfer by NAD(+)-dependent Sir2 histone/protein deacetylases. Proc Natl Acad Sci U S A. 2004;101(23):8563–8. Epub 2004/05/20. doi: 10.1073/pnas.0401057101 . 15150415 10.1073/pnas.0401057101 PMC423234

[52] Yamagata K, Goto Y, Nishimasu H, Morimoto J, Ishitani R, Dohmae N, et al Structural basis for potent inhibition of SIRT2 deacetylase by a macrocyclic peptide inducing dynamic structural change. Structure. 2014;22(2):345–52. Epub 2014/01/07. doi: 10.1016/j.str.2013.12.001 . 24389023 10.1016/j.str.2013.12.001

[53] Long F, Vagin AA, Young P, Murshudov GN. BALBES: a molecular-replacement pipeline. Acta Crystallogr D Biol Crystallogr. 2008;64(Pt 1):125–32. Epub 2007/12/21. doi: 10.1107/S0907444907050172 . 18094476 10.1107/S0907444907050172 PMC2394813

[54] Jin L, Wei W, Jiang Y, Peng H, Cai J, Mao C, et al Crystal structures of human SIRT3 displaying substrate-induced conformational changes. J Biol Chem. 2009;284(36):24394–405. Epub 2009/06/19. doi: 10.1074/jbc.M109.014928 . 19535340 10.1074/jbc.M109.014928 PMC2782032

[55] Bheda P, Jing H, Wolberger C, Lin H. The Substrate Specificity of Sirtuins. Annu Rev Biochem. 2016;85:405–29. Epub 2016/04/19. doi: 10.1146/annurev-biochem-060815-014537 . 27088879 10.1146/annurev-biochem-060815-014537

[56] Parenti MD, Bruzzone S, Nencioni A, Del Rio A. Selectivity hot-spots of sirtuin catalytic cores. Mol Biosyst. 2015;11(8):2263–72. Epub 2015/06/11. doi: 10.1039/c5mb00205b . 26061123 10.1039/c5mb00205b

[57] Rumpf T, Schiedel M, Karaman B, Roessler C, North BJ, Lehotzky A, et al Selective Sirt2 inhibition by ligand-induced rearrangement of the active site. Nat Commun. 2015;6:6263 Epub 2015/02/13. doi: 10.1038/ncomms7263 . 25672491 10.1038/ncomms7263 PMC4339887

[58] Rumpf T, Gerhardt S, Einsle O, Jung M. Seeding for sirtuins: microseed matrix seeding to obtain crystals of human Sirt3 and Sirt2 suitable for soaking. Acta Crystallogr F Struct Biol Commun. 2015;71(Pt 12):1498–510. Epub 2015/12/02. doi: 10.1107/S2053230X15019986 . 26625292 10.1107/S2053230X15019986 PMC4666478

[59] Sundriyal S, Moniot S, Mahmud Z, Yao S, Di Fruscia P, Reynolds CR, et al Thienopyrimidinone Based Sirtuin-2 (SIRT2)-Selective Inhibitors Bind in the Ligand Induced Selectivity Pocket. J Med Chem. 2017;60(5):1928–45. Epub 2017/01/31. doi: 10.1021/acs.jmedchem.6b01690 . 28135086 10.1021/acs.jmedchem.6b01690 PMC6014686

[60] de Cassia Ruy P, Torrieri R, Toledo JS, de Souza Alves V, Cruz AK, Ruiz JC. Intrinsically disordered proteins (IDPs) in trypanosomatids. BMC Genomics. 2014;15:1100 Epub 2014/12/17. doi: 10.1186/1471-2164-15-1100 . 25496281 10.1186/1471-2164-15-1100 PMC4378006

[61] Dyson HJ, Wright PE. Intrinsically unstructured proteins and their functions. Nat Rev Mol Cell Biol. 2005;6(3):197–208. Epub 2005/03/02. doi: 10.1038/nrm1589 . 15738986 10.1038/nrm1589

[62] Peng Z, Mizianty MJ, Xue B, Kurgan L, Uversky VN. More than just tails: intrinsic disorder in histone proteins. Mol Biosyst. 2012;8(7):1886–901. Epub 2012/05/01. doi: 10.1039/c2mb25102g . 22543956 10.1039/c2mb25102g

[63] Gao J, Xu D. Correlation between posttranslational modification and intrinsic disorder in protein. Pac Symp Biocomput. 2012:94–103. Epub 2011/12/17. . 22174266 PMC5120255

[64] Iakoucheva LM, Radivojac P, Brown CJ, O’Connor TR, Sikes JG, Obradovic Z, et al The importance of intrinsic disorder for protein phosphorylation. Nucleic Acids Res. 2004;32(3):1037–49. Epub 2004/02/13. doi: 10.1093/nar/gkh253 . 14960716 10.1093/nar/gkh253 PMC373391

[65] Wong YH, Lee TY, Liang HK, Huang CM, Wang TY, Yang YH, et al KinasePhos 2.0: a web server for identifying protein kinase-specific phosphorylation sites based on sequences and coupling patterns. Nucleic Acids Res. 2007;35(Web Server issue):W588–94. Epub 2007/05/23. doi: 10.1093/nar/gkm322 . 17517770 10.1093/nar/gkm322 PMC1933228

[66] Flick F, Luscher B. Regulation of sirtuin function by posttranslational modifications. Front Pharmacol. 2012;3:29 Epub 2012/03/10. doi: 10.3389/fphar.2012.00029 . 22403547 10.3389/fphar.2012.00029 PMC3289391

[67] Michishita E, Park JY, Burneskis JM, Barrett JC, Horikawa I. Evolutionarily conserved and nonconserved cellular localizations and functions of human SIRT proteins. Mol Biol Cell. 2005;16(10):4623–35. Epub 2005/08/05. doi: 10.1091/mbc.E05-01-0033 . 16079181 10.1091/mbc.E05-01-0033 PMC1237069

[68] Pan M, Yuan H, Brent M, Ding EC, Marmorstein R. SIRT1 contains N- and C-terminal regions that potentiate deacetylase activity. J Biol Chem. 2012;287(4):2468–76. Epub 2011/12/14. doi: 10.1074/jbc.M111.285031 . 22157016 10.1074/jbc.M111.285031 PMC3268407

[69] Fessel MR, Lira CB, Giorgio S, Ramos CH, Cano MI. Sir2-Related Protein 1 from Leishmania amazonensis is a glycosylated NAD+-dependent deacetylase. Parasitology. 2011;138(10):1245–58. Epub 2011/08/09. doi: 10.1017/S0031182011001077 . 21819639 10.1017/S0031182011001077

[70] Cui H, Kamal Z, Ai T, Xu Y, More SS, Wilson DJ, et al Discovery of potent and selective sirtuin 2 (SIRT2) inhibitors using a fragment-based approach. J Med Chem. 2014;57(20):8340–57. Epub 2014/10/03. doi: 10.1021/jm500777s . 25275824 10.1021/jm500777s

