# Supplementary figures and images for: The crystal structure of the Leishmania infantum Silent Information Regulator 2 related protein 1: Implications to protein function and drug design
Source: PLoS One. 2018 Mar 15;13(3):e0193602. doi: 10.1371/journal.pone.0193602 (PMC5854310; doi:10.1371/journal.pone.0193602)

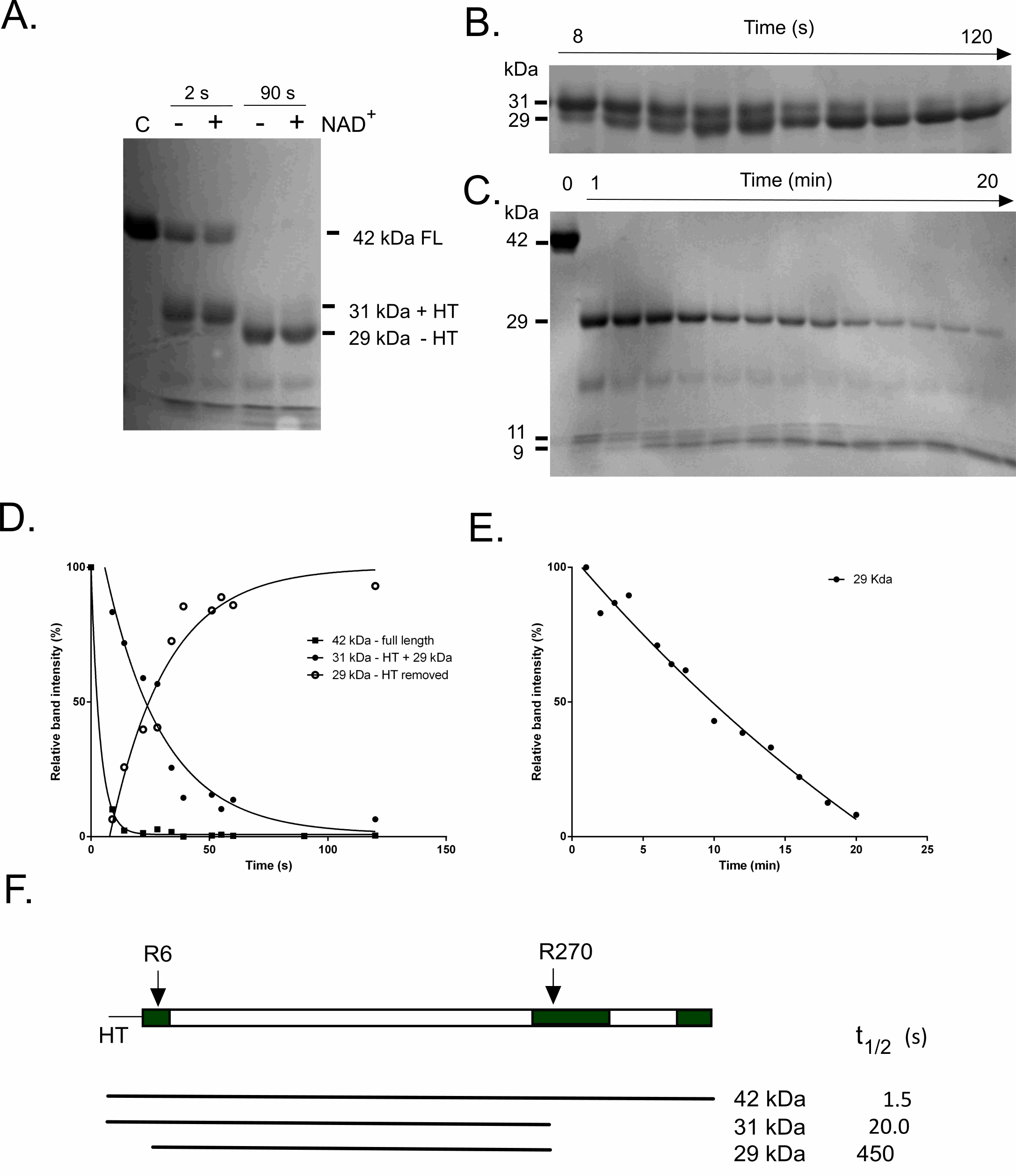

Supplement: S1 Fig — Native trypsin digestion of the hexa histidine tagged LiSIR2rp1 protein was carried out as described for Fig 1 (see Methods). (A) Time resolved native digests showing (i) the rapid loss of full length (42 kDa) protein with concomitant appearance of a His tagged 31 kDa fragment (at 2s) and (ii) the subsequent removal of the N-terminal His tag and the first six LiSIR2rp1 residues from the 31 kDa fragment to yield a stable 29 kDa fragment (at 90s), as described in the text. The presence or absence of NAD+ had no effect on the digestion pattern. (B) and (D) Time resolved digestion and SDS PAGE analysis of the 31kDa to 29 kDa proteolysis (cleavage at R6) and associated quantification of relative band intensities over time. (C) and (E) Time resolved digestion and SDS PAGE analysis of the proteolytic disappearance of the 29 kDa N-terminal fragment and associated quantification of relative band intensity over time. (F) Schematic representation of the data (A-E), showing the principal proteolytic cleavage sites, the corresponding N-terminal fragments, and comparison of their relative half-lives (in seconds). Green bars represent portions of the protein predicted to be disordered (DisEMBL, Remark 465 algorithm) (Linding et al, 2003). Quantitative data was fitted to first order exponential decay [y = A0.exp(-kt)] or association [y = A0 (1- exp(-kt))] models where A0 = 100% (GraphPad prism 6.0 software). (TIF) [file pone.0193602.s001.tif]

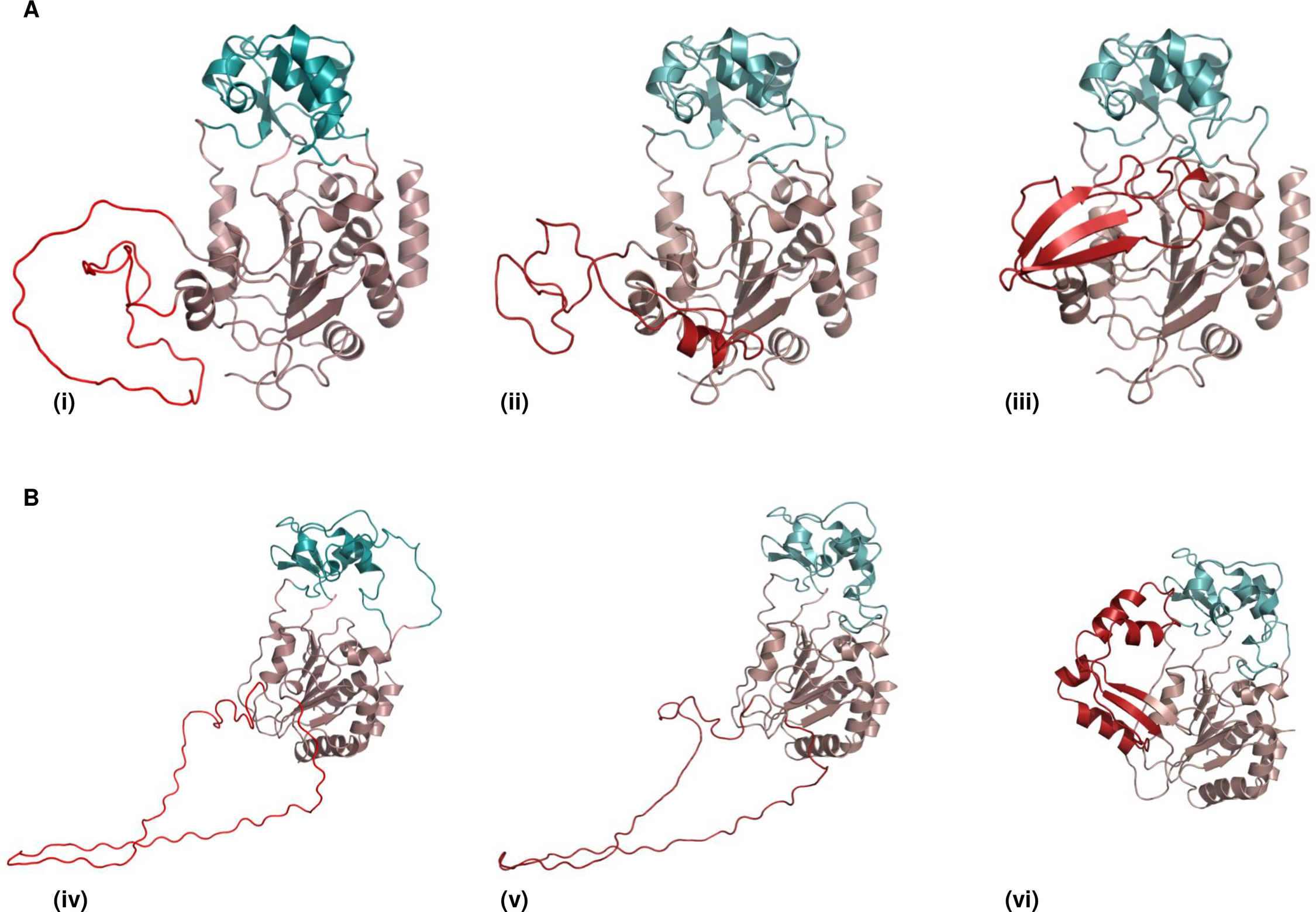

Supplement: S2 Fig — Models (i) and (ii) are based on hSIRT2 pdb 3zgo and model (iii) on hSIRT2 pdb 1j8f. Models (iv), (v) and (vi) are based on ScHst2 pdb 1q14, 1q17 and 1q1a, respectively. The large Rossmann-fold and small zinc-binding domains are colored in turquoise and pink respectively. The LiSIR2RP1 insertion region is colored in red (amino acids 250–300 and 250–320 for human- and yeast-homologue based model respectively). (TIF) [file pone.0193602.s002.tif]

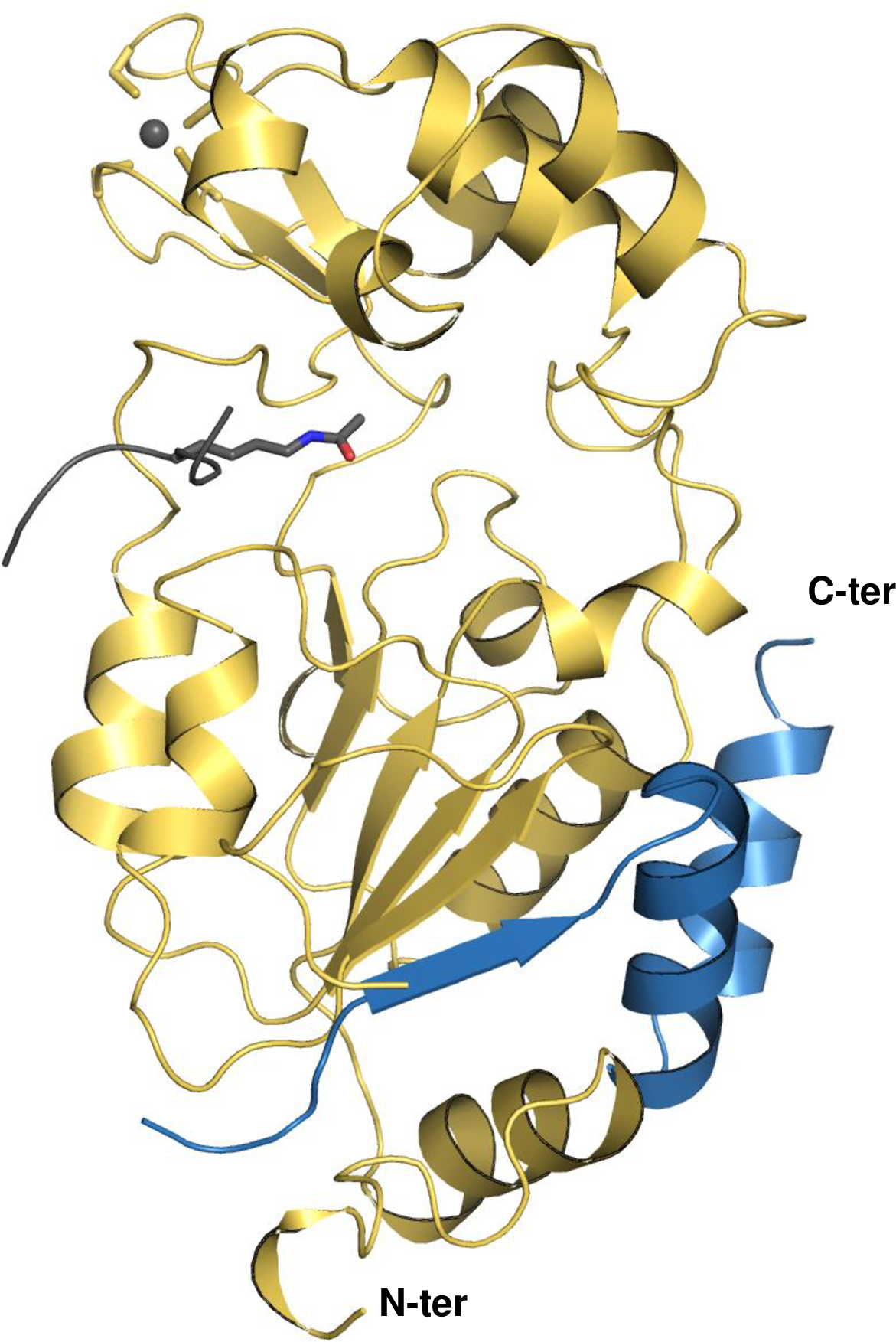

Supplement: S3 Fig — The two proteolytically-stable domains are colored in the same way as Fig 1. The p53 peptide substrate is in grey with acetyl-lysine side chain depicted as sticks. (TIF) [file pone.0193602.s003.tif]

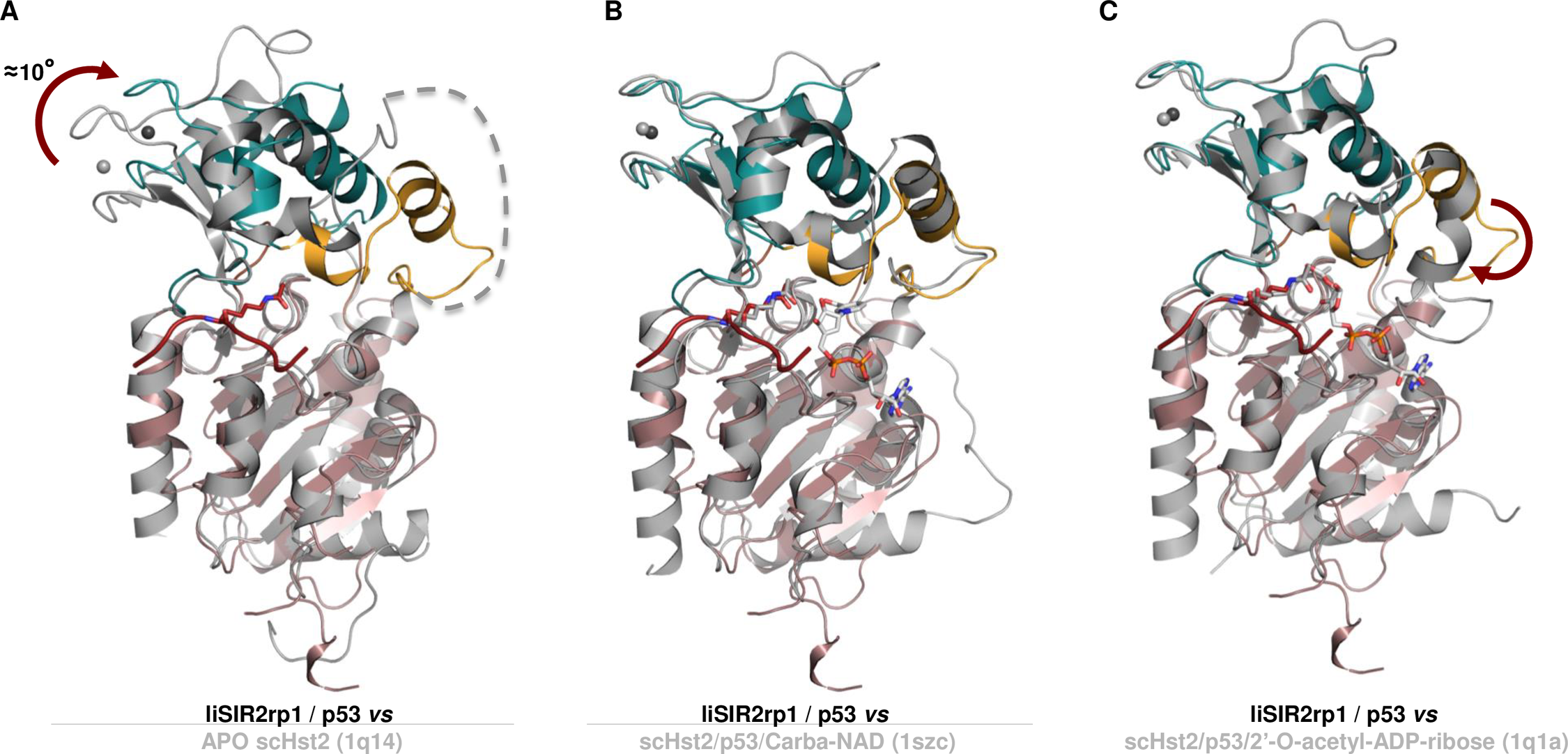

Supplement: S4 Fig — LiSIR2rp1(ΔP253-E303)/p53 is colored as in Fig 3 while ScHst2 is shown in grey. The acetyl-lysine residue, substrate analogue (carba-NAD) and product (2’-O-acetyl-ADP-ribose) are shown as sticks. A. Superposition of LiSIR2rp1(ΔP253-E303)/p53 complex with apo ScHst2 (pdb: 1q14) evidenced a rigid rotation of Leishmania small zinc-binding domain towards the large Rossmann-fold domain (≈10°). The ScHst2 cofactor-binding loop is disordered (dashed line). (ScHst2 C-ter α-helix 13 was removed for clarity). B. The LiSIR2rp1 co-factor binding loop adopts the ordered open conformation as described for ScHst2 in presence of peptide substrate and carba-NAD (pdb: 1zsc). C. When bound to peptide substrate and product, the ScHst2 cofactor binding loop is in a closed conformation. (TIF) [file pone.0193602.s004.tif]
