# Supplementary material for: The crystal structure of the Leishmania infantum Silent Information Regulator 2 related protein 1: Implications to protein function and drug design
Source: PLoS One. 2018 Mar 15;13(3):e0193602. doi: 10.1371/journal.pone.0193602 (PMC5854310; doi:10.1371/journal.pone.0193602)
Supplement: S1 Table — (PDF) [file pone.0193602.s006.pdf]

Organism	UNIPROT	sirtuin	aa's	Da	pI	Pro	Ser	Thr	Sequence of the β8 - β9 connector region *
Leishmania									
Leishmania infantum	Q8I6E4	SIR2rp1	84	9014	4.4	7	16	3	RVGGLLFRFPDDPLNTVHEDAVAKEGRSSSSQSRSPSASPRREEGGTEDSPSSP NEE VEEASTSSSSDGYGQYGDYHAHPDVCR
Leishmania major	Q25337	SIR2rp1	84	8961	4.2	5	16	3	RVGGLLFRFPDDPLDTIHDDAVAKEGRSSSSQSRSPSASARREEGGTEDGSSSP NEEVEDASTSSSSDGYGQYGDYYAHPDVCR
Leishmania amazonensis	Q8IT90	SIR2rp1	84	8819	4.8	5	15	2	RVGGLLFRFPDDPLNTVHDDAVAKEGQSSSSQSRSPSASARCEKGGVEDRSSS PKEGGDEASTSGSSDGYGQYGDYHAHPDLCR
Leptomonas									
Leptomonas pyrrhocoris	A0A0N0VI28	SIR2rp1	103	10389	3.9	6	21	3	RVGGSFFRFPDDDDVPDSAEGLAATSSAASSSSSRPLLAGDGVGADSGDGHRS AAASTAPESIAATRESNEEGREEDAASSSSSSSDGYAQYGDYDVHPPLCR
Leptomonas seymouri	A0A0N1HVQ5	SIR2**	93	9562	3.9	9	14	4	RVGGNLFRFPDDELPVFMDAVGATGAPSSSPPQPPEAAGSADGHSSATGSTP QQCTAGLLEGDVEEREEVSSSSSSDGYAQYGEYSAHPELCR
Phytomonas									
Phytomonas sp. isolate Hart1	W6L9Z1	SIR2**	102	10835	4.2	3	13	3	IFNLERVGGRMFYLPEDVMDEKKASGREDSSSASSAAATHAEDDGIAMNGNA AATLHGNCDFDRNALEEPLLGEVASDSSSSSSEGFITEHEEALLRPAVCR
Trypanosoma									
Trypanosoma brucei brucei	Q57V41	SIR2rp1	63	6825	9.5	2	11	5	RVGGRMFRFPTDRTPNFRASSYRLSTGNGNGSKISSGDSSNSSSVDGYDQFTL AENDETGVLR
Trypanosoma cruzi	Q4DP02	SIR2**	60	6214	7.4	2	17	3	RVGGAMFRFPTDEPTLNSFSRNHTGNSHSSSNSSSSSSSSSSDGYSQFANHAA DAGGICR
Non kinetoplastids									
Plasmodium falciparum	Q8IE47	SIR2A							Region not observed
Toxoplasma gondii	B9PMV3	SIR2	9	962	11.0	0	1	3	TTGLTNRIS
Entamoeba histolytica	M3S670	SIR2**	13	1515	9.7	0	0	0	KVGKMMCYDNLGR
Schistosoma mansoni	T1VXA1	SIRT2	36	4210	10.2	0	5	1	YNDGSTESGLSSFIMRFMVAGFKQNYMKWGRSDNKR
Drosophila melanogaster	Q9VK34	SIR2		1751	5.1	0	1	0	QLHHLKFDVELLGDS
Saccharomyces cerevisiae	P06700	SIR2	7	827	7.8	1	0	0	PVKHAEF
Saccharomyces cerevisiae	P53686	Hst2	12	1334	10.6	1	0	2	TVGDFKANKRPT
Homo sapiens	Q96EB6	SIRT1	7	860	8.0	2	0	0	PLPHLHF
Homo sapiens	Q8IXJ6	SIRT2	28	2979	9.6	1	2	0	KAGQSDPFLGMIMGLGGGMDFDSKKAYR
Homo sapiens	Q9NTG7	SIRT3	12	1389	12.4	2	1	0	LVGPLAWHPRSR

* The connector region between β8 and β9 strands is defined by reference to conserved flanking amino acids Glu250 and Asp263 of ScHst2 via multiple sequence alignments (e.g. equivalent to Glu243 and Asp328 in LiSIR2rp1). ** hypothetical sir2rp1 homologs of ScHst2/hSIRT2.
