# Supplementary material for: The crystal structure of the Leishmania infantum Silent Information Regulator 2 related protein 1: Implications to protein function and drug design
Source: PLoS One. 2018 Mar 15;13(3):e0193602. doi: 10.1371/journal.pone.0193602 (PMC5854310; doi:10.1371/journal.pone.0193602)
Supplement: S2 Table — (PDF) [file pone.0193602.s007.pdf]

Deletion mutant	Primer	Sequence
All	1	5’ CGAATTCCATATGACAGCGTCTCCGAGAGCGCCACAT 3’
	2	5’ CAAGCTTGGATCCTCACGTCTCATTCGGCGCCCTCTG 3’
ΔP253-E303	3	5’ GGCGGCCTCCTCTTCCGCTTTGCGTCGACGTCCAGCTCGAGC 3’
	4	5’ GCTCGAGCTGGACGTCGACGCAAAGCGGAAGAGGAGGCCGCC 3’
ΔP253-H322	5	5’ GGCGGCCTCCTCTTCCGCTTTCCCGATGTCTGCCGGGATGTT 3’
	6	5’ AACATCCCGGCAGACATCGGGAAAGCGGAAGAGGAGGCCGCC 3’
ΔS272-S310	7	5’ GTTGCCAAGGAGGGACGCTCGGACGGCTACGGGCAGTACGGTGAC 3’
	8	5’ GTCACCGTACTGCCCGTAGCCGTCCGAGCGTCCCTCCTTGGCAAC 3’
ΔS272-H322	9	5’ GTTGCCAAGGAGGGACGCTCGCCCGATGTCTGCCGGGATGTT 3’
	10	5’ AACATCCCGGCAGACATCGGGCGAGCGTCCCTCCTTGGCAAC 3’
