# Supplementary material for: The crystal structure of the Leishmania infantum Silent Information Regulator 2 related protein 1: Implications to protein function and drug design
Source: PLoS One. 2018 Mar 15;13(3):e0193602. doi: 10.1371/journal.pone.0193602 (PMC5854310; doi:10.1371/journal.pone.0193602)
Supplement: S3 Table — (PDF) [file pone.0193602.s008.pdf]

| Primer | Sequence |
|---|---|
| 1 | 5’ CGTCTAGAATGACAGCGTCTCC 3’ |
| 2 | 5’ CGCATATGTCACGTCTCATTCG 3’ |
| 3 | 5’ GCGAGAAGCCAGAGATAT 3’ |
| 4 | 5’ TGCAACAGTCGGATGAAG 3’ |
| 5 | 5’ TCTTCACTGGTGTCAATG 3’ |
| 6 | 5’ GACGATACAAGTCAGGTT 3’ |
| 7 | 5’ CGCATACACTATTCTCAGA 3’ |
| 8 | 5’ TCGTTGTCAGAAGTAAGTT 3’ |
| 9 | 5’ AACGACCCCTCAATAC 3’ |
| 10 | 5’ CCTACCATGCCTTCTA 3’ |
