# Supplementary material for: The crystal structure of the Leishmania infantum Silent Information Regulator 2 related protein 1: Implications to protein function and drug design
Source: PLoS One. 2018 Mar 15;13(3):e0193602. doi: 10.1371/journal.pone.0193602 (PMC5854310; doi:10.1371/journal.pone.0193602)
Supplement: S1 Text — (PDF) [file pone.0193602.s009.pdf]

1. Sample description

1.1. SIR2RP1 protein

```
        10         20         30         40         50         60
MARGSHHHHH HLEMTASPRA PHQEHVLGEP TLEGLAHYIR EKNVRRILVL VGAGASVAAG

        70         80         90        100        110        120
IPDFRSPDTG IYANLGKYNL EDPTDAFSLT LLREKPEIFY SIARELNLWP GHFQPTAVHH

       130        140        150        160        170        180
FIRLLQDEGR LLRCCTQNID GLEKAAGVSP ELLVEAHGSF AAAACIECHT PFSIEQNYLE

       190        200        210        220        230        240
AMSGTVSRCS TCGGIVKPNV VFFGENLPDA FFDALHHDAP IAELVIIIGT SMQVHPFALL

       250        260        270        280        290        300
PCVVPKSVPR VVMNRERVGG LLFRFPDDPL NTVHEDAVAK EGRSSSSQSR SPSASPRREE

       310        320        330        340        350        360
GGTEDSPSSP NEEVEEASTS SSSDGYGQYG DYHAHPDVCR DVLFRGDCQE NVVTLAEYLG

       370        380
LSEALAKRMR LSDAAPATAQ RAPNET
```

Molecular weight = 42112.13

Residues = 386

Isoelectric Point = 5.7

Extinction Coefficient = 17210

2. Experimental section

2.1. LC-UV/MS

HPLC system: Alliance 2695 (Waters)

UV detector: PDA 2996 (Waters)

Column	XBRIDGE BEH300 C4 3.5µm (2.1×50mm)
Column temperature	50°C
Flow rate	0.2 ml/min
Gradient	5% to 90% B in 30 min
Mobile phase A	0.05% TFA/0.1% FA/Water
Mobile phase B	0.045% TFA/0.1% FA/70% ACN/30% iPrOH
Diode Array Detection	200-400 nm

HPLC system was coupled to ESI-TOF mass spectrometer (LCT, Waters) equipped with the Triversa Nanomate (Advion) microfluidic nano-ESI device. Mass spectrometer was operated in the positive ion mode in the mass range m/z 500-5000.

2.2. Peptide assignment

BioLynx was used to assign the protein cleavage products detected by LC-UV/MS. Search criteria included trypsin digestion specificity, mass tolerance was set at 0.6 Da for peptides smaller than 10kDa, and at 1 Da for large protein products (> 20kDa).

3. Results

LC-UV analysis of SIR2RP1 submitted to limited trypsin digestion provided LC profile depicted in figure 1. Two very low intensity peaks (①) eluted first from the BEH column (8.99 and 9.53 min). Two major peaks, ② and ③, eluted at 14.84 min and 20.29 min, respectively.

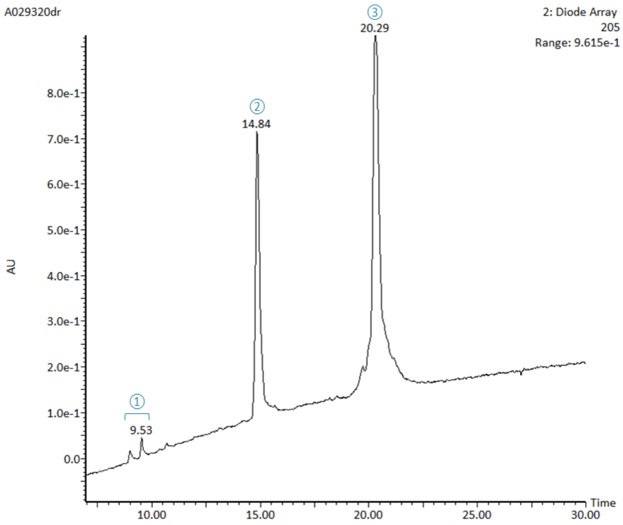

Figure 1: LC elution profile of SIR2RP1 sample following limited trypsin proteolysis.

MS analysis revealed a number of species eluting in the UV-detected peaks:

UV peaks	MS detected species	ESI mass spectrum
①	A : 4619.08 Da	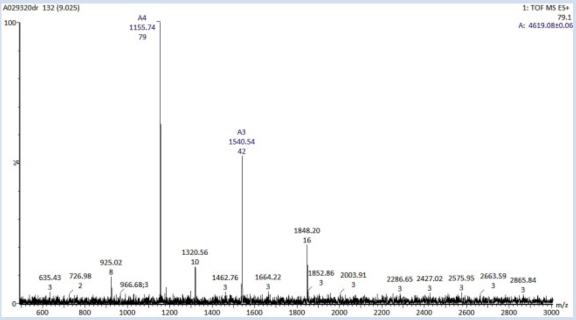

UV peaks	MS detected species	ESI mass spectrum
②	A : 7710.43 Da<br>B : 7999.25 Da<br>C : 9081.40 Da<br>D : 9593.98 Da	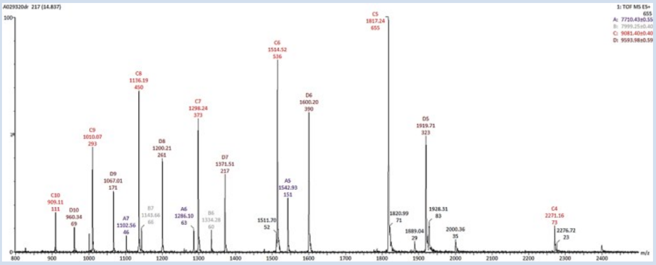
③	A : 28580.42 Da<br>B : 28921.53 Da	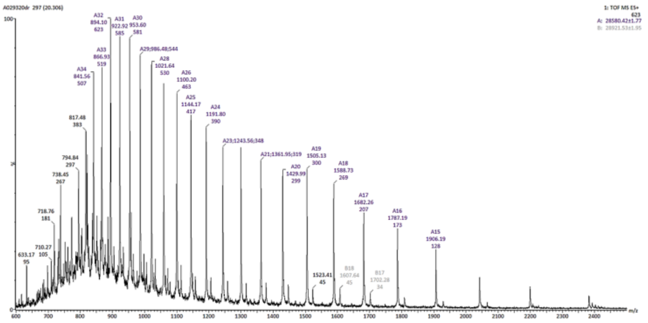

Peptide assignment was performed as described in the experimental section unless specified otherwise in the table below.

Peak ①

Species "A" : 4619.08 Da

Mass tolerance: 0.60
Number of hits = 1

Hit#	Res#	Mass	Sequence	C-Term	Total Mass	Dev	S-S
▪ 1	298-340	4619.61	(R) REEGGTEDSPSSPNEEV EEASTSSSSDGYGQYGDYHA HPDVCR (D)	-OH	4619.61	0.53	-

```
1    MARGS HHHHH HLEMT ASPRA PHQEH VLGEP TLEGL AHYIR
41   EKNVR RILVL VGAGA SVAAG IPDFR SPDTG IYANL GKYNL
81   EDPTD AFSLT LLREK PEIFY SIARE LNLWP GHFQP TAVHH
121  FIRLL QDEGR LLRCC TQNID GLEKA AGVSP ELLVE AHGSF
161  AAAAC IECHT PFSIE QNYLE AMSGT VSRCS TCGGI VKPNV
201  VFFGE NLPDA FFDAL HHDAP IAELV IIIGT SMQVH PFALL
241  PCVVP KSVPR VVMNR ERVGG LLFRF PDDPL NTVHE DAVAK
281  EGRSS SSQSR SPSAS PRREE GGTED SPSSP NEEVE EASTS
321  SSSDG YGQYG DYHAH PDVCR DVLFR GDCQE NVVTL AEYLG
361  LSEAL AKRMR LSDAA PATAQ RAPNE T
```

Peak ②

Species "A" : 7710.43 Da

First peptide mass search with done with a mass tolerance of 0.6 Da and trypsin specificity provided no results. Mass tolerance was then increased to 2 Da while keeping trypsin digestion specificity. With these search criteria, a single peptide hit was found but would assume one disulfide bridge to explain the unusually large mass error (1.71 Da).

Mass tolerance: 2.00
Number of hits = 3

Hit#	Res#	Mass	Sequence	C-Term	Total Mass	Dev	S-S
▪ 3	298-368	7712.14	(R) REEGGTEDSPSSPNEEV EEASTSSSSDGYGQYGDYHA HPDVCRDVLFRGDCQENVVT LAEYLGLSEALAKR (M)	-OH	7712.14	1.71	-

```
1    MARGS HHHHH HLEMT ASPRA PHQEH VLGEP TLEGL AHYIR
41   EKNVR RILVL VGAGA SVAAG IPDFR SPDTG IYANL GKYNL
81   EDPTD AFSLT LLREK PEIFY SIARE LNLWP GHFQP TAVHH
121  FIRLL QDEGR LLRCC TQNID GLEKA AGVSP ELLVE AHGSF
161  AAAAC IECHT PFSIE QNYLE AMSGT VSRCS TCGGI VKPNV
201  VFFGE NLPDA FFDAL HHDAP IAELV IIIGT SMQVH PFALL
241  PCVVP KSVPR VVMNR ERVGG LLFRF PDDPL NTVHE DAVAK
281  EGRSS SSQSR SPSAS PRREE GGTED SPSSP NEEVE EASTS
321  SSSDG YGQYG DYHAH PDVCR DVLFR GDCQE NVVTL AEYLG
361  LSEAL AKRMR LSDAA PATAQ RAPNE T
```

Species "B" : 7999.25 Da

Mass tolerance: 0.60
Number of hits = 1

Hit#	Res#	Mass	Sequence	C-Term	Total Mass	Dev	S-S
▪ 1	298-370	7999.52	(R) REEGGTEDSPSSPNEEV EEASTSSSSDGYGQYGDYHA HPDVCRDVLFRGDCQENVVT LAEYLGLSEALAKRMR (L)	-OH	7999.52	0.27	-

```
1    MARGS HHHHH HLEMT ASPRA PHQEH VLGEP TLEGL AHYIR
41   EKNVR RILVL VGAGA SVAAG IPDFR SPDTG IYANL GKYNL
81   EDPTD AFSLT LLREK PEIFY SIARE LNLWP GHFQP TAVHH
121  FIRLL QDEGR LLRCC TQNID GLEKA AGVSP ELLVE AHGSF
161  AAAAC IECHT PFSIE QNYLE AMSGT VSRCS TCGGI VKPNV
201  VFFGE NLPDA FFDAL HHDAP IAELV IIIGT SMQVH PFALL
241  PCVVP KSVPR VVMNR ERVGG LLFRF PDDPL NTVHE DAVAK
281  EGRSS SSQSR SPSAS PRREE GGTED SPSSP NEEVE EASTS
321  SSSDG YGQYG DYHAH PDVCR DVLFR GDCQE NVVTL AEYLG
361  LSEAL AKRMR LSDAA PATAQ RAPNE T
```

Peak ②

Species “C” : 9081.40 Da

Mass tolerance: 0.60
Number of hits = 2

Hit#	Res#	Mass	Sequence	C-Term	Total Mass	Dev	S-S
▪ 2	298-381	9081.71	(R) REEGGTEDSPSSPNEEV<br>EEASTSSSSDGYGQYGDYHA<br>HPDVCRDVLFRGDCQENVVT<br>LAEYLGLSEALAKRMRLSDA<br>APATAQR (A)	-OH	9081.71	0.31	-

```
1     MARGS HHHHH HLEMT ASPRA PHQEH VLGEP TLEGL AHYIR
41    EKNVR RILVL VGAGA SVAAG IPDFR SPDTG IYANL GKYNL
81    EDPTD AFSLT LLREK PEIFY SIARE LNLWP GHFQP TAVHH
121   FIRLL QDEGR LLRCC TQNID GLEKA AGVSP ELLVE AHGSF
161   AAAAC IECHT PFSIE QNYLE AMSGT VSRCS TCGGI VKPNV
201   VFFGE NLPDA FFDAL HHDAP IAELV IIIGT SMQVH PFALL
241   PCVVP KSVPR VVMNR ERVGG LLFRF PDDPL NTVHE DAVAK
281   EGRSS SSQSR SPSAS PRREE GGTED SPSSP NEEVE EASTS
321   SSSDG YGQYG DYHAH PDVCR DVLFR GDCQE NVVTL AEYLG
361   LSEAL AKRMR LSDAA PATAQ RAPNE T
```

Species “D” : 9593.98 Da

Mass tolerance: 0.60
Number of hits = 1

Hit#	Res#	Mass	Sequence	C-Term	Total Mass	Dev	S-S
▪ 1	298-386	9594.23	(R) REEGGTEDSPSSPNEEV<br>EEASTSSSSDGYGQYGDYHA<br>HPDVCRDVLFRGDCQENVVT<br>LAEYLGLSEALAKRMRLSDA<br>APATAQRAPNET (-)	-OH	9594.23	0.25	-

```
1     MARGS HHHHH HLEMT ASPRA PHQEH VLGEP TLEGL AHYIR
41    EKNVR RILVL VGAGA SVAAG IPDFR SPDTG IYANL GKYNL
81    EDPTD AFSLT LLREK PEIFY SIARE LNLWP GHFQP TAVHH
121   FIRLL QDEGR LLRCC TQNID GLEKA AGVSP ELLVE AHGSF
161   AAAAC IECHT PFSIE QNYLE AMSGT VSRCS TCGGI VKPNV
201   VFFGE NLPDA FFDAL HHDAP IAELV IIIGT SMQVH PFALL
241   PCVVP KSVPR VVMNR ERVGG LLFRF PDDPL NTVHE DAVAK
281   EGRSS SSQSR SPSAS PRREE GGTED SPSSP NEEVE EASTS
321   SSSDG YGQYG DYHAH PDVCR DVLFR GDCQE NVVTL AEYLG
361   LSEAL AKRMR LSDAA PATAQ RAPNE T
```

Peak ③

Species “A” : 28580.43 Da

Mass tolerance: 1.00
Number of hits = 1

Hit#	Res#	Mass	Sequence	C-Term	Total Mass	Dev	S-S
▪ 1	20-280	28579.86	(R) APHQEHVLGEPTLEGLA<br>HYIREKNVRRILVLVGAGAS<br>VAAGIPDFRSPDTGIYANLG<br>KYNLEDPTDAFSLTLLREKP<br>EIFYSIARELNLWPGHFQPT<br>AVHHFIRLLQDEGRLLRCCT<br>QNIDGLEKAAGVSPELLVEA<br>HGSFAAAACIECHTPFSIEQ<br>NYLEAMSGTVSRCSTCGGIV<br>KPNVVFFGENLPDAFFDALH<br>HDAPIAELVIIIGTSMQVHP<br>FALLPCVVPKSVPRVVMNRE<br>RVGGLLFRFPDDPLNTVHED<br>AVAK (E)	-OH	28579.86	-0.57	-

```
1     MARGS HHHHH HLEMT ASPRA PHQEH VLGEP TLEGL AHYIR
41    EKNVR RILVL VGAGA SVAAG IPDFR SPDTG IYANL GKYNL
81    EDPTD AFSLT LLREK PEIFY SIARE LNLWP GHFQP TAVHH
121   FIRLL QDEGR LLRCC TQNID GLEKA AGVSP ELLVE AHGSF
161   AAAAC IECHT PFSIE QNYLE AMSGT VSRCS TCGGI VKPNV
201   VFFGE NLPDA FFDAL HHDAP IAELV IIIGT SMQVH PFALL
241   PCVVP KSVPR VVMNR ERVGG LLFRF PDDPL NTVHE DAVAK
281   EGRSS SSQSR SPSAS PRREE GGTED SPSSP NEEVE EASTS
321   SSSDG YGQYG DYHAH PDVCR DVLFR GDCQE NVVTL AEYLG
361   LSEAL AKRMR LSDAA PATAQ RAPNE T
```

Species “B” : 28921.53 Da

Mass tolerance: 1.00
Number of hits = 1

Hit#	Res#	Mass	Sequence	C-Term	Total Mass	Dev	S-S
▪ 1	20-283	28922.21	(R) APHQEHVLGEPTLEGLA<br>HYIREKNVRRILVLVGAGAS<br>VAAGIPDFRSPDTGIYANLG<br>KYNLEDPTDAFSLTLLREKP<br>EIFYSIARELNLWPGHFQPT<br>AVHHFIRLLQDEGRLLRCCT<br>QNIDGLEKAAGVSPELLVEA<br>HGSFAAAACIECHTPFSIEQ<br>NYLEAMSGTVSRCSTCGGIV<br>KPNVVFFGENLPDAFFDALH<br>HDAPIAELVIIIGTSMQVHP<br>FALLPCVVPKSVPRVVMNRE<br>RVGGLLFRFPDDPLNTVHED<br>AVAKEGR (S)	-OH	28922.21	0.68	-

```
1     MARGS HHHHH HLEMT ASPRA PHQEH VLGEP TLEGL AHYIR
41    EKNVR RILVL VGAGA SVAAG IPDFR SPDTG IYANL GKYNL
81    EDPTD AFSLT LLREK PEIFY SIARE LNLWP GHFQP TAVHH
121   FIRLL QDEGR LLRCC TQNID GLEKA AGVSP ELLVE AHGSF
161   AAAAC IECHT PFSIE QNYLE AMSGT VSRCS TCGGI VKPNV
201   VFFGE NLPDA FFDAL HHDAP IAELV IIIGT SMQVH PFALL
241   PCVVP KSVPR VVMNR ERVGG LLFRF PDDPL NTVHE DAVAK
281   EGRSS SSQSR SPSAS PRREE GGTED SPSSP NEEVE EASTS
321   SSSDG YGQYG DYHAH PDVCR DVLFR GDCQE NVVTL AEYLG
361   LSEAL AKRMR LSDAA PATAQ RAPNE T
```
