# Supplementary material for: The crystal structure of the Leishmania infantum Silent Information Regulator 2 related protein 1: Implications to protein function and drug design
Source: PLoS One. 2018 Mar 15;13(3):e0193602. doi: 10.1371/journal.pone.0193602 (PMC5854310; doi:10.1371/journal.pone.0193602)
Supplement: S2 Text — (PDF) [file pone.0193602.s010.pdf]

Design of LiSIR2RP1 deleted mutants

Attempts to crystallize full length LiSIR2RP1 alone or in complex with peptide substrate and/or cofactor were unsuccessful. This led us to the design of deletion mutants of the protein based on detailed *in silico* analysis of LiSIR2RP1 sequence (including multiple sequence alignments with human and yeast SIRT2 homologues, disordered/globular domain and secondary structure predictions and LiSIR2RP1 homology structure generation).

Compared to successfully crystallized homologues (Finnin et al., 2001; Zhao et al., 2003a; Zhao et al., 2003b; Zhao et al., 2004; Moniot et al., 2013; Yamagata, 2014), multiple sequence alignment provided evidence for an insertion region in LiSIR2RP1 (region ≈270-325) that could impact formation of crystals. In the design of internal deletion mutants, limits of deletion had to be determined in order to remove maximum of the LiSIR2rp1 insertion region while keeping enough residues to maintain the global fold of the protein, not to disrupt any secondary structure elements and limit structural constraints.

According to NPS@ and PSIPRED algorithms, LiSIR2rp1 insertion region is flanked by two α-helices (≈260-270 and 325-330, respectively). In LiSIR2rp1 ΔS272-S310 and ΔS272-H322 mutants, this region with no or few secondary structure elements is removed keeping a linker of approximately 17 and 5 residues (for ΔS272-S310 and ΔS272-H322, respectively) between the two flanking predicted helices. Sequences kept to form the linkers were also designed in order to exclude maximum of the multiple serine stretches found in the LiSIR2rp1 insertion region.

Design of LiSRI2RP1 ΔP253-E303 and ΔP253-H322 were mainly based on homology 3D models generated with human and yeast SIRT2 homologues respectively (SWISS-MODEL). The two typical domains found in Sirtuins: the Rossman-fold domain and the small Zn-binding domain each comprising the main Sirtuins' structural features are present in all the generated models. The misaligned region, from residue 250 to 300 for the human homologue-based model and 250 to 320 for the yeast homologue-based model, was removed in the deletion mutants. The length of the linkers was commensurate to the distance separating the two secondary structure elements of the Rossman fold between

which LiSIR2rp1 additional region inserts, thus limiting structural constraints that may impair global fold of LiSIR2rp1.

References

Finnin MS, Donigian JR, Pavletich NP. Structure of the histone deacetylase SIRT2. Nat Struct Biol. 2001;8(7):621-5.

Moniot S, Schutkowski M, Steegborn C. Crystal structure analysis of human Sirt2 and its ADP-ribose complex. J Struct Biol. 2013;182(2):136-43.

Yamagata K, Goto Y, Nishimasu H, Morimoto J, Ishitani R, Dohmae N, et al. Structural basis for potent inhibition of SIRT2 deacetylase by a macrocyclic peptide inducing dynamic structural change. Structure. 2014;22(2):345-52.

Zhao K, Chai X, Clements A, Marmorstein R. Structure and autoregulation of the yeast Hst2 homolog of Sir2. Nat Struct Biol. 2003;10(10):864-71.

Zhao K, Chai X, Marmorstein R. Structure of the yeast Hst2 protein deacetylase in ternary complex with 2'-O-acetyl ADP ribose and histone peptide. Structure. 2003;11(11):1403-11.

Zhao K, Harshaw R, Chai X, Marmorstein R. Structural basis for nicotinamide cleavage and ADP-ribose transfer by NAD(+)-dependent Sir2 histone/protein deacetylases. Proc Natl Acad Sci U S A. 2004;101(23):8563-8.
