# Supplementary material for: The crystal structure of the Leishmania infantum Silent Information Regulator 2 related protein 1: Implications to protein function and drug design
Source: PLoS One. 2018 Mar 15;13(3):e0193602. doi: 10.1371/journal.pone.0193602 (PMC5854310; doi:10.1371/journal.pone.0193602)
Supplement: S3 Text — (PDF) [file pone.0193602.s011.pdf]

DisEMBL™

Prediction results for Q8I6E4_LEIIN

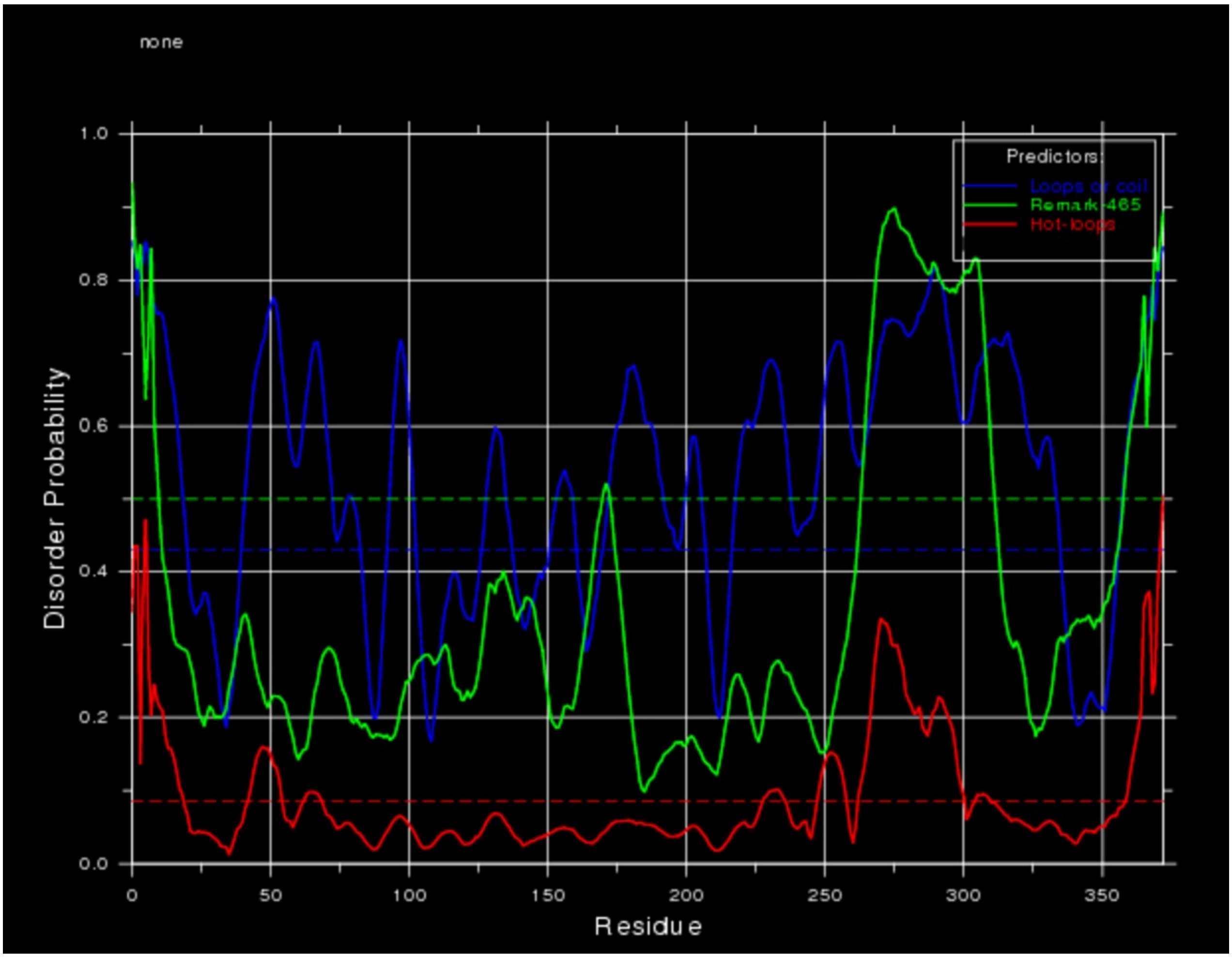

[Download PostScript file]

Disordered by Loops/coils definition

```
> Q8I6E4_LEIIN_LOOPS 1-20, 41-83, 94-103, 128-138, 152-161, 171-207, 218-335, 357-373
MTASPRAPHQ EHVLGEPTLE glahyirekn vrrilvlvga GASVAAGIPD FRSPDTGIYA NLGKYNLEDP TDAFSLTLLR
EKPeifysia relNLWPGHF QPTavhhfir llqdegrllr cctqnidGLE KAAGVSPEll veahgsfaaa aCIECHTPFS
Ieqnyleams GTVSRCSTCG GIVKPNVVFF GENLPDAFFD ALHHDAPiae lviiigtSMQ VHPFALLPCV VPKSVPRVVM
NRERVGGLLF RFPDDPLNTV HEDAVAKEGR SSSSQSRSPS ASPRREEGGT EDSPSSPNEE VEEASTSSSS DGYGQYGDYH
AHPDVCRDVL FRGDCqenvv tlaeylglse alakrmRLSD AAPATAQRAP NET
```

Disordered by Hot-loops definition

```
> Q8I6E4_LEIIN_HOTLOOPS 1-19, 44-55, 249-258, 263-301, 360-373
MTASPRAPHQ EHVLGEPTLe glahyirekn vrrilvlvga gasVAAGIPD FRSPDtgiya nlgkynledp tdafsltllr
ekpeifysia relnlwpghf qptavhhfir llqdegrllr cctqnidgle kaagvspell veahgsfaaa aciechtpfs
ieqnyleams gtvsrcstcg givkpnvvff genlpdaffd alhhdapiae lviiigtsmq vhpfallpcv vpksvprvvm
nrervgglLF RFPDDPLNtv heDAVAKEGR SSSSQSRSPS ASPRREEGGT EDSPSSPNEE Veeastssss dgygqygdyh
ahpdvcrdvl frgdcqenvv tlaeylglse alakrmrlsD AAPATAQRAP NET
```

Disordered by Remark-465 definition

```
> Q8I6E4_LEIIN_REM465 1-10, 264-312, 359-373
MTASPRAPHQ ehvlgeptle glahyirekn vrrilvlvga gasvaagipd frspdtgiya nlgkynledp tdafsltllr
ekpeifysia relnlwpghf qptavhhfir llqdegrllr cctqnidgle kaagvspell veahgsfaaa aciechtpfs
ieqnyleams gtvsrcstcg givkpnvvff genlpdaffd alhhdapiae lviiigtsmq vhpfallpcv vpksvprvvm
nrervggllf rfpddplntv hedAVAKEGR SSSSQSRSPS ASPRREEGGT EDSPSSPNEE VEEASTSSSS DGygqygdyh
ahpdvcrdvl frgdcqenvv tlaeylglse alakrmrlSD AAPATAQRAP NET
```

**JOB-ID**	Q8I6E4_LEIIN_164640WgSGcAoLCBIAAH45I88AAAAB
**Frames used**	smooth=8 peak=8 join=4
**Thresholds used**	coils=0.516 rem465=0.6 hot loops=0.1204
**Name**	Q8I6E4_LEIIN
**Description**	none
**Title/ID**	none
**Sequence length**	373
**Download predictions**	smoothed scores raw scores

DisEMBL™ is Copyright © 2003-2006 by Rune Linding & Lars Juhl Jensen - EMBL
