# Supplementary material for: The crystal structure of the Leishmania infantum Silent Information Regulator 2 related protein 1: Implications to protein function and drug design
Source: PLoS One. 2018 Mar 15;13(3):e0193602. doi: 10.1371/journal.pone.0193602 (PMC5854310; doi:10.1371/journal.pone.0193602)
Supplement: S4 Text — (PDF) [file pone.0193602.s012.pdf]

DISPHOS Results

> LiSIR2rp1
MTASPRAPHQEHVLGEPTLEGLAHYIREKNVRRILVLVGAGASVAAGIPDFRSPDTGIYANLGKYN
LEDPTDAFSLTLLREKPEIFYSIARELNLWPGHFQPTAVHHFIRLLQDEGRLLRCCTQNIDGLEKA
AGVSPELLVEAHGSFAAAACIECHTPFSIEQNYLEAMSGTVSRCSTCGGIVKPNVVFFGENLPDAF
FDALHHDAPIAELVIIIGTSMQVHPFALLPCVVPKSVPRVVMNRERVGGLLFRFPDDPLNTVHEDA
VAKEGRSSSSQSRSPSASPRREEGGTEDSPSSPNEEVEEASTSSSSDGYGQYGDYHAHPDVCRDVLF
RGDCQENVVTLAEYLGLSEALAKRMRLSDAAPATAQRAPNET

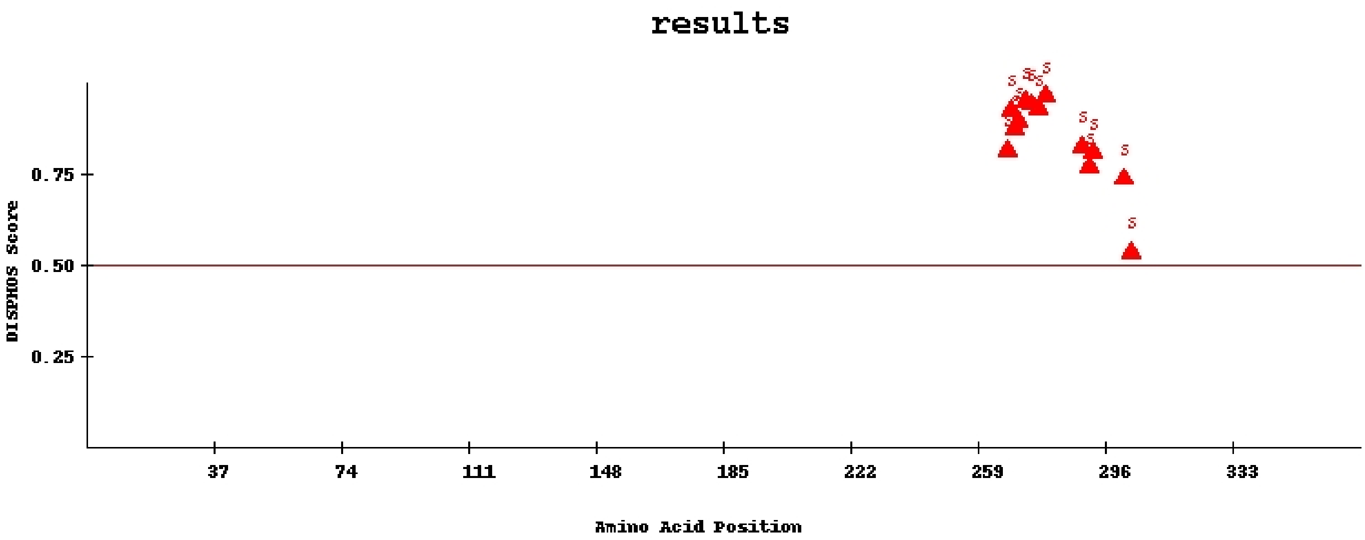

Statistics
Number of phosphorylated serines: 13 out of 31 (41.935%)
Number of phosphorylated threonines: 0 out of 17 (0.000%)
Number of phosphorylated tyrosines: 0 out of 9 (0.000%)

Position	Residue	Score	Sequence	Yes/No
2	T	0.095	***MTASPR	
4	S	0.180	*MTASPRAP	
18	T	0.036	LGEPTLEGL	
25	Y	0.041	GLAHYIREK	
43	S	0.051	GAGASVAAG	
53	S	0.091	PDFRSPDTG	
56	T	0.023	RSPDTGIYA	
59	Y	0.128	DTGIYANLG	
65	Y	0.044	NLGKYNLED	
71	T	0.017	LEDPTDAFS	
75	S	0.059	TDAFSLTLL	
77	T	0.005	AFSLTLLRE	

87	Y	0.029	PEIFYSIAR	
88	S	0.081	EIFYSIARE	
103	T	0.027	HFQPTAVHH	
123	T	0.005	LRCCTQNID	
136	S	0.100	AAGVSPELL	
146	S	0.073	EAHGSFAAA	
157	T	0.014	IECHTPFSI	
160	S	0.026	HTPFSIEQN	
165	Y	0.024	IEQNYLEAM	
170	S	0.052	LEAMSGTVS	
172	T	0.005	AMSGTVSRC	
174	S	0.034	SGTVSRCST	
177	S	0.030	VSRCSTCGG	
178	T	0.020	SRCSTCGGI	
217	T	0.003	IIIGTSMQV	
218	S	0.008	IIGTSMQVH	
234	S	0.102	VVPKSVPRV	
259	T	0.114	DPLNTVHED	
271	S	0.821	KEGRSSSSQ	YES
272	S	0.932	EGRSSSSQS	YES
273	S	0.878	GRSSSSQSR	YES
274	S	0.899	RSSSSQSRS	YES
276	S	0.955	SSSQSRSPS	YES
278	S	0.947	SQSRSPSAS	YES
280	S	0.934	SRSPSASPR	YES
282	S	0.969	SPSASPRRE	YES
290	T	0.294	EEGGTEDSP	
293	S	0.831	GTEDSPSSP	YES
295	S	0.774	EDSPSSPNE	YES
296	S	0.814	DSPSSPNEE	YES
305	S	0.742	VEEASTSSS	YES
306	T	0.065	EEASTSSSS	
307	S	0.541	EASTSSSSD	YES
308	S	0.480	ASTSSSSDG	
309	S	0.481	STSSSSDGY	
310	S	0.346	TSSSSDGYG	

313	Y	0.483	SSDG**Y**GQYG
316	Y	0.216	GYGQ**Y**GDYH
319	Y	0.165	QYGD**Y**HAHP
341	T	0.004	ENVV**T**LAEY
345	Y	0.031	TLAE**Y**LGLS
349	S	0.109	YLGL**S**EALA
359	S	0.389	RMRL**S**DAAP
365	T	0.279	AAPA**T**AQRA
373	T	0.051	APNE**T******

*An asterisk indicates prediction by similarity to the training sequences
